# Heterogeneous Ensemble Combination Search Using Genetic Algorithm for Class Imbalanced Data Classification

**DOI:** 10.1371/journal.pone.0146116

**Published:** 2016-01-14

**Authors:** Mohammad Nazmul Haque, Nasimul Noman, Regina Berretta, Pablo Moscato

**Affiliations:** 1 The Priority Research Centre for Bioinformatics, Biomarker Discovery and Information-Based Medicine, Hunter Medical Research Institute, New Lambton Heights, New South Wales, Australia; 2 School of Electrical Engineering and Computer Science, Faculty of Engineering and Built Environment, The University of Newcastle, Callaghan, New South Wales, Australia; University of Ulm, GERMANY

## Abstract

Classification of datasets with imbalanced sample distributions has always been a challenge. In general, a popular approach for enhancing classification performance is the construction of an ensemble of classifiers. However, the performance of an ensemble is dependent on the choice of constituent base classifiers. Therefore, we propose a genetic algorithm-based search method for finding the optimum combination from a pool of base classifiers to form a heterogeneous ensemble. The algorithm, called GA-EoC, utilises 10 fold-cross validation on training data for evaluating the quality of each candidate ensembles. In order to combine the base classifiers decision into ensemble’s output, we used the simple and widely used majority voting approach. The proposed algorithm, along with the random sub-sampling approach to balance the class distribution, has been used for classifying class-imbalanced datasets. Additionally, if a feature set was not available, we used the (*α*, *β*) − *k* Feature Set method to select a better subset of features for classification. We have tested GA-EoC with three benchmarking datasets from the UCI-Machine Learning repository, one Alzheimer’s disease dataset and a subset of the PubFig database of Columbia University. In general, the performance of the proposed method on the chosen datasets is robust and better than that of the constituent base classifiers and many other well-known ensembles. Based on our empirical study we claim that a genetic algorithm is a superior and reliable approach to heterogeneous ensemble construction and we expect that the proposed GA-EoC would perform consistently in other cases.

## Introduction

An Ensemble of Classifiers (EoC) is a collection of trained classifier models whose predictions are combined to reach a final decision. According to the Wolpert’s *no free lunch theorem* [1], one classifier may perform well in some domains, but never in all application domains. Therefore, by combining the outputs of many classifiers, the ensemble of classifiers strategically enhances the power of the committee, as an aggregated method, to achieve better prediction accuracy than any of the individual classifiers could alone [2]. Moreover, the generalisation capability of each classifier also differs depending on the data characteristics and relations (such as dimensionality, class distributions, noise ratio and so on) [3]. In recent years, many difficult “real-world” datasets have been characterised as imbalanced [4], where sample distributions among classes are skewed. This characteristic is common in data arising in diverse domains: fraud detection, intrusion detection, medical diagnosis and monitoring, bioinformatics, text categorisation, image processing are only few examples. Traditional classification algorithms are biased to the overrepresented class and produce an unacceptably low classification rate for the minority class [5]. Over the last decade, ensemble-based classification systems have remained the centre of attention, gaining popularity, and demonstrating their applicability for class imbalanced data [6–8]. Although every EoC combines multiple classifier outcomes into a single decision, their building paradigms usually differ in the diversity generation mechanism among the base classifiers and the strategy of combining them.

Diversity among the base classifiers is one of the key issues in ensemble formation. Since some base classifiers may make mistakes in different instances, strategically combining them can reduce the total error [9]. The most practised diversity generation mechanism is the *homogeneous ensemble*, in which different distributions of the original training dataset are used to train various instances of one base classifier. Bagging and boosting [10–12] are two well-known homogeneous ensemble methods. These ensemble methods could be biased to some specific characteristics of the dataset because of their training using a single type of base classifier. The use of different base classifiers to create an EoC is another approach for introducing diversity and is referred to as a *heterogeneous ensemble*. These methods can be advantageous for learning different characteristics of the training dataset, since they use a diverse set of base classifiers.

The next key element in ensemble formation is the combination of base classifiers, which determine how to combine all base classifiers’ outcomes into a final decision. There are numerous combination approaches, such as majority voting, weighted majority voting, summation, product, maximum and minimum, fuzzy integral, Dempster-Shafer based fusion or decision templates [9, 13, 14].

To deal with imbalanced datasets, several homogeneous EoCs have been proposed [5, 15–18]. In addition, oversampling, undersampling, and sampling by synthetically generating some instances are commonly used sampling techniques for improving the classification performance [7]. Very few heterogeneous EoCs have been proposed so far for imbalanced data classification (such as [19, 20]) and those ensembles were built with every base classifiers in the ensemble.

The main objective of an EoC is to achieve better generalisation. However, not all ensemble combinations (even created with all base classifiers) can achieve this goal. It is important to find the optimal ensemble combination (set of base classifiers) for the classification task. The number of possible combinations for ensembles increases exponentially with the number of base classifiers in the pool. Therefore an exhaustive search for the optimal combination is not practical as evaluation of each combination is computationally expensive. Heuristic algorithms are feasible approaches that can help to find a near-optimal solution in a reasonable time. Several of them have been proposed for exploring ensemble combinations both for homogeneous and heterogeneous types.

Margineantu and Dietterich [21] proposed a greedy algorithm for selecting homogeneous ensemble (boosting-based ensemble) using a forward search approach. They added one decision tree base classifier at each step of the search to formulate the ensemble combination. Caruana et al. [22] used greedy algorithms for searching for the best ensemble combination for a heterogeneous ensemble. They generated 2000 instances of base classifiers by varying the parameters of seven classifiers. Then, they added one base classifier at each step into the ensemble combination to maximise the performance. The main drawback of this method is having no way to replace a previously selected based classifier at a later stage. Partalas et al. [23] proposed a heterogeneous ensemble method which used a greedy approach based on the predictive performance of the current combination. They created the ensemble with different learning algorithms using various parameters. The average rank of the proposed method was better when compared to the other approaches used in the experiment. Niculescu et al. [24] won the 2009 KDD Cup Orange Challenge using a homogeneous ensemble found by a greedy algorithm. The inclusion of a base classifier into the decision tree was dependent on its performance on the validation dataset. They changed the base classifiers for different classification problems. For one problem in the competition, they only used the six best classifiers instead of using all selected base classifiers. Recently, Bhatnagar et al. [25] proposed a heuristic method for searching a homogeneous ensemble combination which considered the accuracy of individual classifiers as well as the pairwise diversity amongst those classifiers. It created a ensemble combination using fewer base classifiers by rejecting new base classifiers in the ensemble while the accuracy remained the same. All these studies suggest that greedy heuristics are able to find good EoCs. However, greedy algorithms can easily be stuck in a local optima and more adaptable heuristics should be used in complex search problems like the ensemble combination search.

Genetic algorithms (GA) have also been used for searching for ensemble combinations in some studies. Kim and Oh [26] used a Hybrid Genetic Algorithm (HGA) for searching for a homogeneous ensemble combination. They incorporated two local search operations in their HGA. They trained multiple nearest neighbor classifiers (as base classifiers) using randomly chosen feature subsets from the training dataset. Ekbal and Saha [27] also used a GA to search a homogeneous ensemble combination. The method was applied on named entity recognition datasets and achieved superior classification performance when compared to the best base classifier and two other ensemble methods. Thammasiri and Meesad [28] proposed a GA-based classifier ensemble method. From 3 types of base classifiers, they trained 30 instances by random sampling of training data, similar to how base classifiers are used in homogeneous ensembles. They used majority vote in order to increase the ensemble classification accuracy. The experimental outcome on three small (maximum feature and sample count was 30 and 1000 respectively) datasets from the UCI repository showed that the ensemble combination selected by the GA yielded higher performance than the individual base classifiers and two other ensemble approaches. Note that the GAs cited above have only been used in searching for optimal ensemble combination in homogeneous ensembles ([28] is also homogeneous ensemble depending on the base classifier generation approach). Hence the competency of GAs in searching for heterogeneous ensemble combinations is yet to be explored. Thus is the focus of this work.

In this paper, we propose a GA-based method for selecting the best classifiers to produce a good heterogeneous EoC. The search space is determined by all possible combinations of base classifiers. For each combination, a 10-fold cross validation of the full training data has been used. As a result, it creates a complex search space for finding the best combination of base classifiers. We use the random sub-sampling method for handling class-imbalanced datasets and unweighted majority voting as the fusion mechanism. To solve the search problem, we propose a GA for finding the optimal combination of base classifiers for the ensemble. The best ensemble found by the GA is applied to the test data to evaluating its effectiveness. Since the *Matthews Correlation Coefficient (MCC)* [29] provides a more representative measure for generalisation performance on imbalanced data classification, we considered it as the key performance measure in the proposed method. The proposed GA-based searching for ensemble combination, named GA-EoC, is evaluated using several datasets with imbalanced-class distributions.

## Materials and Methods

In the first part of this section, we define the unweighted majority vote-based ensemble of classifiers (EoC). Each base classifier of the ensemble is allowed to cast a single vote for the class label per sample from the test dataset. The class that achieves majority vote from the base classifiers would be the final class label of the sample. The goal of the proposed algorithm is to maximise the MCC score as the fitness measure of the heterogeneous ensemble of classifiers. In the second part, we describe the different components of our proposed GA-based ensemble method for imbalanced data classification. Finally, in the final part, we describe the datasets used to evaluate the proposed method.

### Ensemble of Classifiers

A *binary classifier* C learns the mapping or decision function of feature set (*R*^*n*^) from the set $D=\left\{x_{1},\cdots ,x_{n}\right\},x_{i}\in R^{n}$ of training samples to the binary class label set *Ω* = {0,1} i.e.:

$$\begin{array}{c} C:R^{n}\to D\Omega . \end{array}$$ (1)

The next task is to apply this trained classifier to labelling the supplied *m* unlabelled samples of *testing dataset* $U=\{u_{1},\cdots ,u_{m}\}$

$$\begin{array}{c} C(u_{j})\to \Omega \end{array}$$ (2)

where the class label (*Ω*) of each samples $u_{j}\in U$ will be associated with one, and only one, label.

Let *k* be the number of individual base classifiers in the ensemble $E=\langle C_{1},\cdots ,C_{k}\rangle$ trained on the same training dataset D. The unweighted majority voting ensemble classifier outcome for each sample $\left(u_{j}\in U\right)$ is defined as:

$$\begin{array}{c} E(u_{j})=\{\begin{array}{cc} 1 & \;\sum_{i=1}^{k}C_{i}(u_{j})>\frac{k}{2}\\ 0 & \;\sum_{i=1}^{k}C_{i}(u_{j})<\frac{k}{2}\\ \text{Random}\{1,0\} & \;\text{Otherwise} \end{array} \end{array}$$ (3)

The end result of the ensemble of classifiers is produced by the unweighted majority voting by all *k* trained base classifiers.

For a binary classification problem, four possible outcomes arise and are summarised in a 2 × 2 contingency table or confusion matrix [30] and different measures of performance can be calculated from it (sensitivity, specificity and accuracy are the most common measures). We select the Matthews Correlation Coefficient (MCC) as the measure of classification quality. This can be computed from the confusion matrix as

$$\begin{array}{c} MCC=\frac{\left(TP\times TN)-(FP\times FN\right)}{\sqrt{\left(TP+FP)(TP+FN)(TN+FP)(TN+FN\right)}} \end{array}$$ (4)

where *TP*, *FP*, *TN*, *FN* denote the true and false positive and negative values, respectively. The MCC quantifies the strength of the classifications by considering all four outcomes of the confusion matrix. It can often provide a more balanced accuracy assessment of the model [31], even for the imbalanced datasets [32, 33]. Therefore, we consider the MCC as our measure of classification performance.

### The Genetic Algorithm-based EoC (GA-EoC)

A *Genetic Algorithm (GA)* is an optimisation algorithm inspired by natural selection, or “survival of the fittest”. The main difference between GAs and many traditional optimisation methods is that GAs work with a population of solutions rather than a single solution. The implementation of a GA can be parallelised easily because of this multipoint searching characteristic. Moreover a GA is less susceptible to getting stuck in local optima when compared with many other heuristics. The crossover and mutation operations help the heuristic to escape from local optimal solutions by producing significant randomness in the population. These advantages make GAs appropriate for large and complex optimisation problems.

**Fig 1:**
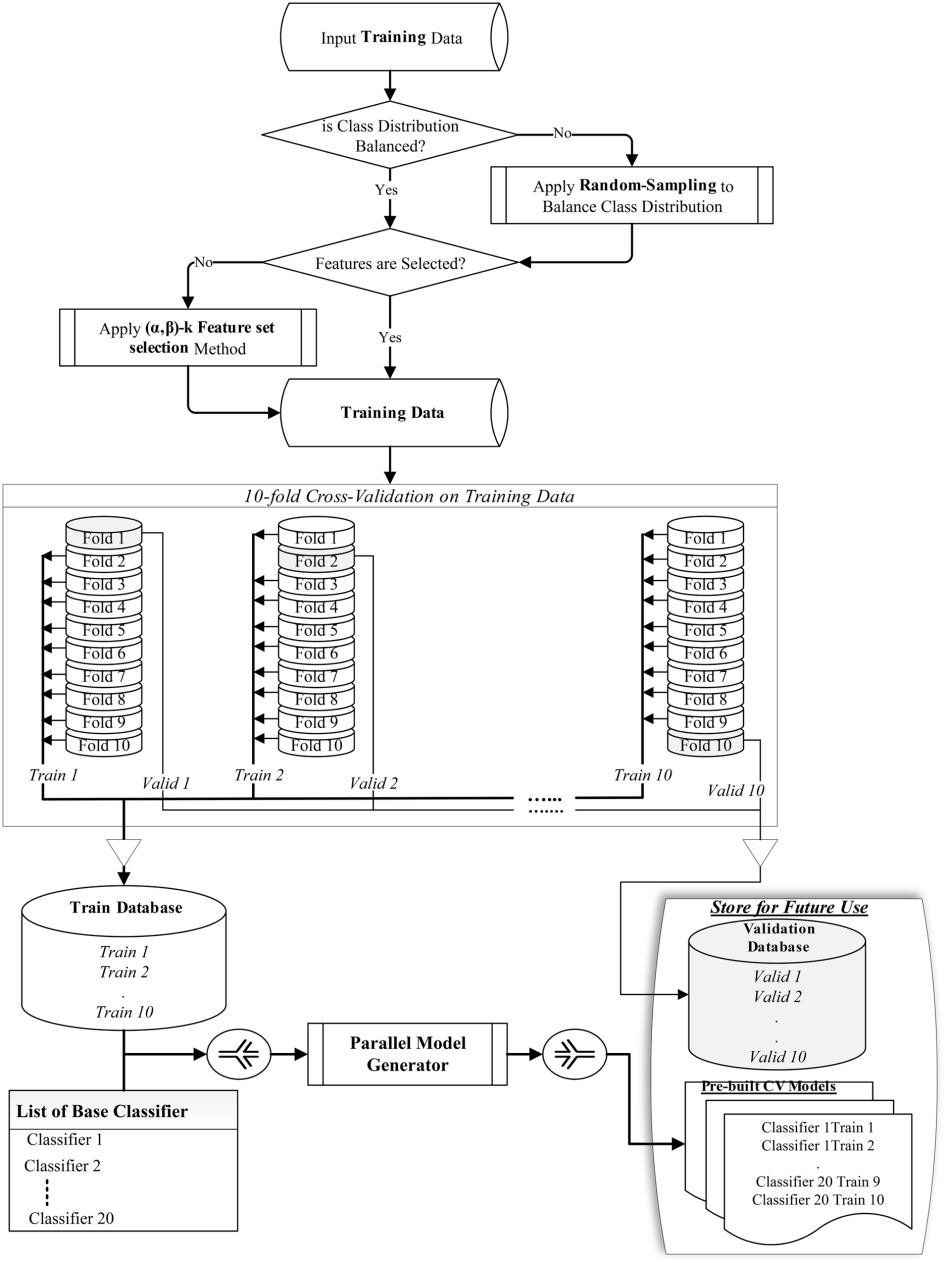
The steps in preprocessing the training dataset and generating the base classifier models. The process starts taking the training dataset as input. First, it balances the class distribution for imbalanced training data. Next, it selects features using (*α*, *β*) − *k* Feature Set selection method if features are not available. Then, it creates train and validation folds from the training dataset for 10-fold cross validation. These folds of the dataset are saved and used for internal validation of ensembles. Finally, it generates the models for each classifiers on each training fold (*Train 1* to *Train 10*) and save them for future use.

**Fig 2:**
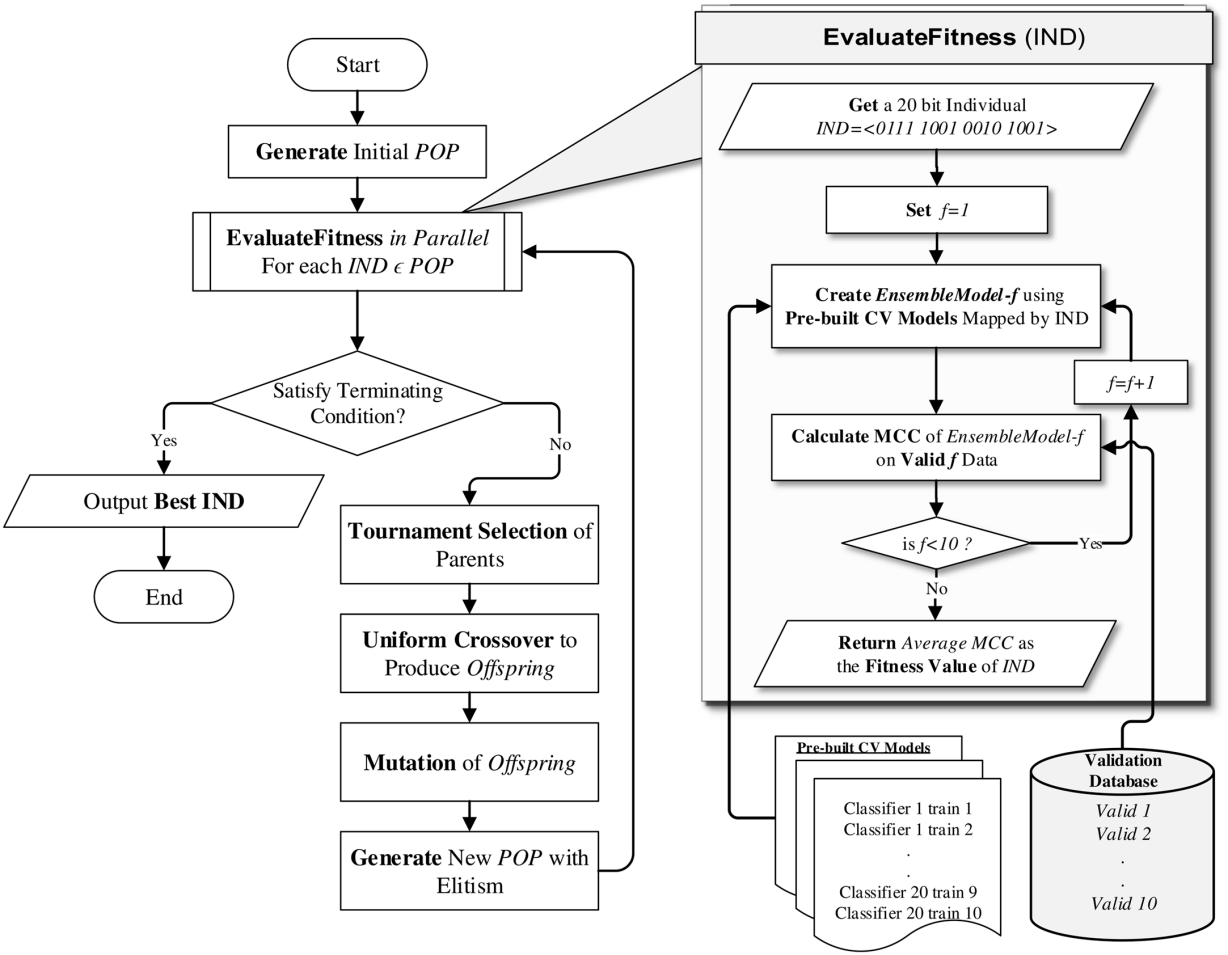
Overall process flow of the proposed GA-EoC algorithm. In GA-EoC, each individual represents an EoC and the genetic algorithm is used to find the best EoC based on its performance on validation folds. For each individual an EoC is constructed using the base classifier models of a training fold and the MCC score of the EoC is calculated for the corresponding validation fold generated beforehand (Fig 1). The average MCC score calculated over 10 folds is taken as the fitness value of the individual. The algorithm iterates creating a new population from the current one until a terminating condition is satisfied. The individual with the best fitness value form the final population is returned as the solution.

**Fig 3:**
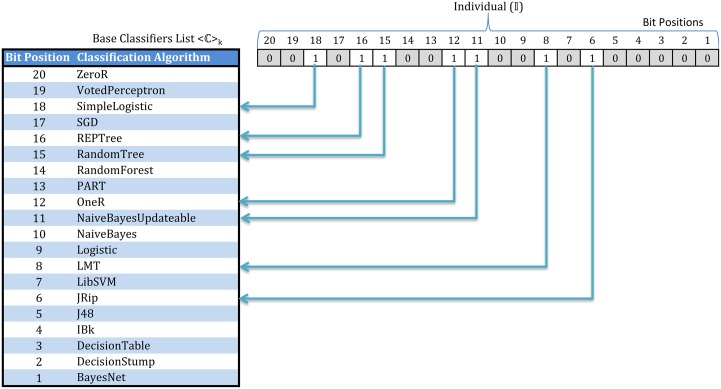
Representation of an individual in GA-EoC and its mapping into the corresponding base classifiers for ensemble combination.

In our design, we chose 20 base classifiers (listed in Table 1) from the Waikato Environment for Knowledge Analysis (WEKA) data mining software suite [34] to create the ensemble combinations. Our chosen 20 classifiers can produce over a million unique ways to create ensembles. Since it is impractical to perform an exhaustive search through this huge set of combinations to find the best ensemble, we have implemented a genetic algorithm to perform a heuristic search. The working process of the whole algorithm is divided into two phases. The first step creates cross validation folds and models on training folds to be used by next phase (Fig 1). Then, the GA is used to search for the best ensemble of classifiers (EoC) from all possible ensemble combinations. This GA-based ensemble of classifiers searching method will be denoted as *GA-EoC* (Fig 2). The working phases of GA-EoC are:

1. **Preprocessing**: In the initial phase, the whole training dataset is taken as input. If the class distribution of the training dataset is imbalanced, we balance the class distribution. Then, we create the training and testing folds for the internal validation process of the GA-EoC using 10-fold cross validation on the training dataset. A detail process description is as follows:
  - Class Distribution Balancing: For a class imbalanced dataset, we keep all samples from the minority class (*Ω*_*min*_) separated. Then we randomise the rest of the dataset containing only majority class labels (*Ω*_*maj*_) and split it into equal proportions to the number of samples in the minority class. Later we consider each portion from the *Ω*_*maj*_ class samples combined with *Ω*_*min*_ class samples to formulate multiple *balanced binary-class* datasets. If the class distribution of the training dataset is balanced, then we skip this step.
  - Feature Selection: If features of the dataset have not been already selected, then we apply the (*α*, *β*) − *k* Feature Set method [35] on the training dataset. The (*α*, *β*) − *k* Feature Set approach (proposed by Cotta et al. [35]) finds a minimum set of features that conjointly maximise the inter-class discrimination and intra-class equity. This method has been applied successfully in several studies for feature selection and biomarker discovery [36–41]. Features selected by the (*α*, *β*) − *k* Feature Set method are kept for further processing of the training dataset. If the features are available then we skip this step and use the given features for classification.
  - 10-fold Cross Validation Dataset Preparation: The training dataset is split for the 10-fold cross validation (CV) method, where the training dataset is randomised and divided into 10 equal sized subsamples or folds. Out of these 10 folds, one is preserved as the validation data, and the other 9 folds are used as training data. This cross-validation process is repeated 10 times, with each of the 10 folds used exactly once as the validation data. Each training and validation split of 10-fold CV is preserved in separate databases named *train database* and *validation database*, respectively (see Fig 1).
2. **Model Generation**: The next phase of the proposed algorithm involves generating the base classifier models. In this phase, only train data (consisting of 9 folds from the training dataset) from the train database are explored (see Fig 1). The process trains each participating base classifier with each of the train data and saves those models for future use. In this way, a total of 200 models are generated in parallel for 20 base classifiers.
3. **The GA-EoC**: The genetic algorithm-based search for finding the best ensemble combination takes place in this phase and it is showed in Fig 2. Next we explain the main elements of our GA-EoC.
  - Individual Representation: We have used *binary encoding* for representing each individual, where each position represents a particular classifier. The selection of a specific classifier depends on the value of corresponding position in the individual. In Fig 3, we have a list ${\langle C\rangle }_{k}=\left\{C_{1},\cdots ,C_{k}\right\}$ of *k* = 20 base classifiers mapped into to a 20-bit individual (I), called an ensemble of classifiers. The mapping function for the individual (I) to an ensemble of classifiers combination (E) is denoted by
    $$\begin{array}{c} I\mapsto E=\langle I\left[i\right]\overset{\equiv 1}{⟶}C_{i}\in {\left\langle C\right\rangle }_{k}\rangle \end{array}$$ (5)
    where, an individual (I) is represented by *k* bit array. Each element of the array I[i] selects the classifier $C_{i}$ from the classifiers list ${\langle C\rangle }_{k}$, if and only if the *i*-th bit position contains a 1 (I[i]≡1 where *i* = 1, ⋯, *k*). These selected classifiers form the ensemble represented by an individual of the genetic algorithm.
  - Population: The GA-EoC begins with a population containing a set of random individuals. The initial population size can be set depending on the problem. A good rule of thumb for determining the size of initial population |P| is given by [42] as
    $$\begin{array}{c} \left|P\right|=min((5\times k),(\frac{1}{2}\times e)) \end{array}$$ (6)
    where *k* is the size of an individual and *e* is the maximum number of possible ensemble combinations (*e* = 2^*k*^). According to the formula, we have created a population containing 100 individuals.
  - Fitness Evaluation: The fitness value of each $I_{i}\in P$ is calculated as follows. The individual $I_{i}$ is mapped to the combination of base classifiers E according to Eq (5). The fitness function (*fit*) for individual I is given by
    $$\begin{array}{c} fit(I)=\frac{1}{10}\sum_{f=1}^{10}MCC\left\langle E\;\text{for}\;\text{fold}f\right\rangle \end{array}$$ (7)
    The fitness evaluation process has been depicted in the method named **EvaluateFitness** in Fig 2. We calculate the fitness value of an ensemble combination (E) using an unweighted majority voting approach as per Eq (3). The ensemble combination is evaluated on each fold; we create the unweighted majority vote ensemble using pre-generated base classifier models for each fold and test its classification performance (according to the MCC metric) against the corresponding validation data taken from the validation database. We repeat this process for each of the 10-folds and the average MCC score is treated as the *fitness value* of the individual.
    This task is performed in parallel, helping to reduce the running time for the fitness calculation process of individuals in the population. Our objective is to find the best individual from the population with the maximum fitness value. The objective function is given by
    $$\begin{array}{c} obj=\underset{i=1,\cdots ,n}{\text{arg max}}\;fit\left(I_{i}\in P\right) \end{array}$$ (8)
    where the function *obj* returns the best individual from the population which *maximises* the fitness value. This denotes the goodness-of-fit measure for individuals in the population and the individual is denoted as *fittest individual*.
  - Creating a New Population: After evaluation of each individual’s fitness, the next task is to generate a new population. The new population generation process involves application of three operations named *selection*, *crossover* and *mutation*. We apply an *elitism* strategy, where the *m* best (in the proposed method we used *m* = 1) individuals are promoted to the new generation without any variation. Now we will briefly describe the processes used for the parent selection and offspring creation.
    - Parent Selection: We used *tournament* selection method for selecting parents for offspring generation. We create a pool of 10 randomly selected individuals from the existing population. Then, the best individual based on the fitness score has been chosen as the first parent for breeding a new individual. We repeat the same process to select a second parent. The role of parent selection is to distinguish among individuals and prefer better individuals as parents of the next generation.
    - Crossover: The purpose of the crossover or recombination operator is to breed a new offspring from a pair of parents. We applied *uniform crossover* which facilitates the mixture of two parents by generates a new offspring from them. After selecting two parents according to the parent selection scheme, each gene (bit) of the offspring is inherited randomly from either of the two parents with a fixed crossover rate (*R*_*χ*_). In this work we applied uniform crossover with a rate of 60%.
    - Mutation: We have applied *random bit replacement* with a mutation rate (*R*_*μ*_). In order to sustain a genetic diversity in the population, we have selected the following mutation rate according to [42]
      $$\begin{array}{c} R_{\mu }=max(0.01,\frac{1}{n}) \end{array}$$ (9)
      where *n* is the population size and *R*_*μ*_ is the mutation rate. So, in the proposed algorithm, we use 0.01 as mutation rate (*R*_*μ*_).
  - Terminating Conditions: We have used three terminating conditions for the algorithm. The algorithm finishes the searching if any of the following conditions is satisfied:
    - The total number of generations has reached 1000.
    - The fitness of the best individual of the population has remained stationary for 50 consecutive generations.
    - The fitness value has reached the global optimal value (MCC is equals to 1.0).

**Table 1:** List of base classifiers used in GA-EoC.

Classifier	Category
BayesNet	Bayes Network
NaiveBayes	Bayes Network
NaiveBayesUpdateable	Bayes Network
LibSVM	Function
Logistic	Function
SGD	Function
SimpleLogistic	Function
VotedPerceptron	Function
IBk	K-NN
DecisionTable	Rule Based
JRip	Rule Based
OneR	Rule Based
PART	Rule Based
RandomTree	Rule Based
REPTree	Rule Based
ZeroR	Rule Based
DecisionStump	Tree
J48	Tree
RandomForest	Tree
LMT	Tree

The genetic algorithm returns the best individual from the training data, which has the best fitness score. Then, we create the ensemble combination model of the best individual and evaluate it as follows. We train the selected base classifiers using the full training dataset and formulate a unweighted majority vote ensemble. The generalisation performance of this ensemble combination is measured by considering the testing dataset classification outcomes. That performance can be compared to other state-of-the-art classification algorithms, including other ensemble-based ones, trained and tested with same training and testing data.

## Results

### Datasets

To evaluate the performance of the proposed method, we chose datasets from the UCI-ML repository [43], one biological dataset on Alzheimer’s disease [44] and a real world dataset for the face recognition problem [45]. Two of the datasets have imbalanced class distribution characteristics. Next the datasets are described in detail.

#### UCI-ML Repository Datasets

We used three datasets from the UCI-ML repository (Table 2). The first one is the “Wisconsin Breast Cancer (Original)”, referred to as *WBC* [46]. This breast cancer dataset contains 9 attributes/features and a total of 699 samples, divided into two classes, named *benign* and *malignant*. The class distribution for this dataset is imbalanced at a ratio of 1.90, where 458 samples are from the *benign* class, and the rest of the 241 samples represent the *malignant* class. The second one is the “Pima Indian diabetes” (*PIMA*) dataset which contains two classes, 8 features and 768 samples. Among these 768 samples, 500 of the tests (about 65.1%) expressed negative results, and 268 samples (about 34.9%) confirmed positive results for diabetes. The class distribution of this dataset is skewed at a ratio of nearly 1.87. We have taken another dataset called *BUPA*. This data set has the information of some liver disorders that might arise from excessive alcohol consumption by individual persons. It contains a total of 345 samples described by 7 features of individual alcohol consumption behaviour divided into two classes. The class-imbalanced ratio for this dataset is 1.38.

**Table 2:** Characteristics of the datasets used for experiments.

Short Name	Name of the Dataset	#Samples (class dist.)	#Features
WBC	Wisconsin Breast Cancer (Original)	699 (458,241)	9
PIMA	Pima Indians Diabetes	768 (500,268)	8
BUPA	BUPA Liver Disorders Data Set	345 (145,200)	7
Ray-AD-Trn-18	Ray et.al.—AD (18 Protein)	83 (43,40)	18
RMoscato-AD-Trn-5	Ravetti Moscato—AD (5 Protein)	83 (43,40)	5
TestSetAD	Ray et.al.—AD (Testing)	92 (42,50)	120
TestSetMCI	Ray et.al.—MCI (Testing)	47 (22,25)	120

#### Alzheimer’s Disease Datasets

We also use a biological Alzheimer’s disease (AD) datasets used in Ray et al. [44]. The dataset contained the signalling protein abundances from blood plasma that could classify Alzheimer’s disease (AD). They measured the abundance of 120 known signalling proteins from 259 archived plasma samples collected from individuals with pre-symptomatic to late-stage AD. The Alzheimer’s and nondemented control (NDC) samples were divided equally into a training set for supervised classification and a test set for class prediction of blinded samples (*Ray-AD-Trn* and *TestSetAD* in Table 2). The authors proposed 18 proteins as biomarkers for classification of AD. The classification was also performed on samples from two previously published cohorts of mild cognitive impairment (MCI) patients (named *TestSetMCI*) who converted to AD, developed other dementias (OD) or remained unchanged at a later stage. They combined the NDC and OD classification data into one group which represented the class of patients who did not convert to Alzheimer’s disease (non-AD). None of the samples from this test set were used in the training process of the classifier.

Ravetti and Moscato [47] applied the (*α*, *β*) − *k* feature selection method for protein biomarker selection on the same dataset. They reported a set of 5-proteins as a better biomarker set (referred as *RMoscato-AD-Trn-5*) for predicting the AD. Their discovered 5-proteins biomarker produce high accuracy in the prediction of Alzhemier’s disease from both testing datasets (*TestSetAD* and *TestSetMCI*).

We used training sets *Ray-AD-Trn-18* and *RMoscato-AD-Trn-5* from both studies to train the proposed GA-EoC separately and test the prediction on *TestSetAD* and *TestSetMCI*. The classification outcomes are compared against both studies.

#### Face Recognition Dataset

The last dataset used to evaluate the GA-EoC was a subset of the *PubFig*83 dataset [45]. Chiachia et al. [48] selected 100 images of each for a group of celebrities and separated them into training and testing sets of 90 and 10 images respectively. They tested a SVM classifier and identified the 5 most difficult celebrities to recognise (shown in Table 3). This subset of the *PubFig*83 dataset contains 450 image samples (90 per class) in the training set. The testing dataset contains a total of 50 image samples (10 samples per class). This subset of the *PubFig*83 dataset (denoted as *PubFig*05) has been used for further processing.

**Table 3:** Distribution of the training and testing data in *PubFig*05 dataset.

Person	class_id	#Train Images	#Test Images
Jenifer Lopez	0	90	10
Katherine Heigl	1	90	10
Scarlett Johansson	2	90	10
Mariah Carey	3	90	10
Jessica Alba	4	90	10
**Total Samples**		**450**	**50**

To extract features from images, the *HT-L3-model* (described in [49]) has been used, yielding 25600 features. Then, we applied an implementation of Fayyad and Irani’s [50] entropy-based filtering method to discretise the training dataset and discard features using the minimum description length (MDL) principle; only 4878 passed this entropy based filtering. Since the proposed method is only able to handle binary-class problems at this stage, we need to convert the dataset to binary-class problem. We separated the *PubFig*05 dataset into 5 binary-class datasets by *one-vs-all* approach (one for each class). These datasets became *imbalanced* at a ratio of 1: 4 and using the procedure described in preprocessing, for each imbalanced dataset, we produced 4 balanced ones, finishing with 20 datasets (4 balanced datasets for each of the 5 classes).

Next, for each class (using the respective 4 balanced binary-class datasets), we applied each procedure described below and the *PubFig*05 turned into 15 datasets, 3 for each class, as shown in Table 4.

1. We apply (*α*, *β*) − *k* Feature Set method on each of the balanced binary-class datasets and take the **union** of all selected features. Then, we apply (*α*, *β*) − *k* Feature Set method on this consolidated binary-class dataset (denoted by *UAB* in Table 4).
2. We apply (*α*, *β*) − *k* Feature Set method similar to the *UAB*. However, instead of taking the union of selected features, we take the **intersection** of them to consolidate into binary-class datasets (denoted by *IAB* in Table 4).
3. In this last procedure, we apply first the entropy filtering on each of the balanced binary-class datasets before applying the (*α*, *β*) − *k* Feature Set method. Then we take the *union* of selected features to consolidate into binary-class dataset. (denoted by (*UEAB* in Table 4).

**Table 4:** Outcome of the (*α*, *β*) − *k* Feature Set selection method for three different setups (UAB, IAB, UEAB) showing the number of selected features per binary-class datasets of *PubFig*05.

Binary Class—Dataset	*UAB*	*IAB*	*UEAB*
Class 0 vs All	4656	4495	795
Class 1 vs All	4702	4598	1554
Class 2 vs All	4712	4563	2273
Class 3 vs All	4678	4501	2821
Class 4 vs All	4738	4553	1081
**Average Features**	**4697**	**4542**	**1705**

Outcomes of these procedures are summarised in Table 4. Details about the procedures and their step by step outcomes are in Tables A, B and C in S1 File and these data are available for public access at http://dx.doi.org/10.5281/zenodo.33539.

### Experimental Results

In this section, we report the results of the experiments performed with the GA-EoC on the above mentioned datasets. For UCI-ML repository datasets, we use 10-fold cross validation (CV). Other datasets used in this study have their separate training and test data for performance evaluation. We used both MCC and accuracy as performance indices and compared the results achieved by the base classifiers and the proposed GA-EoC algorithm. We also contrasted the performance of GA-EoC with that of three popular ensemble methods, namely bagging, boosting and random forest under the same experimental settings. Due to the stochastic nature of the GA-EoC algorithm, each experiment was repeated 100 times except for *PubFig05* dataset in which the experiments were performed only once because of the very high computational time requirement.

The GA-EoC algorithm was implemented in Java. We used the implementations of base classifiers from the Weka framework version 3.6. All the experiments except those on *PubFig05* dataset were executed in Dell PowerEdge III with Dual Xeon 5550 2.67 GHz (8 cores) and 32 GB RAM. The machine was running on Red Hat Enterprise Linux AS release 4 operating system. We executed the experiments for *PubFig05* dataset on Xenon Radon 6170 Supermicro server with Quad E5-4650 Sandy Bridge 2.7GHz (32 cores) and 512GB RAM because of the high memory requirement. The source code of GA-EoC is available at https://sourceforge.net/projects/geneticensembleclassifier/ and other relevant information is available in S2 File.

#### Classification Performances on UCI-ML Repository Datasets

For all of the three datasets from UCI-ML repository the features were available, therefore we did not apply the (*α*, *β*) − *k* Feature Set selection phase for these datasets. However, the class-imbalanced characteristic of these datasets necessitates the application of class distribution balancing phase. After class rebalancing, the cross validation was performed using GA-EoC and the performance metrics were calculated.

The classification performance of the base classifiers and GA-EoC in terms of MCC and accuracy are presented in Tables 5 and 6, respectively. For UCI-ML datasets, we observe that the proposed method achieved an average MCC score of 0.99, 0.94 and 0.50 for WBC, PIMA and BUPA respectively (Table 5). The closest performing base classifiers are the BayesNet classifier with 0.94 MCC score for WBC dataset, the SGD classifier with 0.50 MCC score for the PIMA dataset and the LMT classifier with 0.41 MCC score for BUPA dataset. The MCC gap between the best base classifier and the proposed GA-EoC is at least 0.05 for the datasets taken from UCI-ML repository.

**Table 5:** Classification performances (in MCC scale) of the base classifiers and GA-EoC for all experiments.

Classifier	WBC	PIMA	BUPA	AD-18	MCI-18	AD-5	MCI-5	UAB	IAB	UEAB
BayesNet	0.94	0.43	0.04	0.79	0.31	0.91	0.28	0.37	0.36	0.46
DecisionStump	0.84	0.37	0.20	0.80	0.15	0.80	0.15	0.21	0.11	0.21
DecisionTable	0.87	0.38	0.14	0.80	0.10	0.80	0.10	0.24	0.17	0.22
IBk	0.90	0.33	0.24	**0.89**	0.37	0.79	0.16	0.59	0.60	0.57
J48	0.89	0.42	0.33	**0.89**	0.19	0.80	0.27	0.29	0.30	0.29
JRip	0.89	0.43	0.32	0.59	0.37	0.85	0.10	0.44	0.24	0.31
LibSVM	0.91	0.00	0.13	0.85	0.40	0.87	0.37	0.53	0.52	0.63
LMT	0.91	0.48	0.41	0.76	0.41	0.89	0.53	**0.65**	0.63	0.59
Logistic	0.92	0.48	0.35	0.72	0.41	0.89	0.51	0.49	0.49	0.38
NaiveBayes	0.91	0.47	0.15	0.87	0.33	0.91	0.35	0.39	0.36	0.41
NaiveBayesUpdateable	0.91	0.47	0.15	0.87	0.33	0.91	0.35	0.39	0.36	0.41
OneR	0.84	0.33	0.09	0.80	0.15	0.80	0.15	0.17	0.09	0.17
PART	0.87	0.43	0.26	0.81	0.32	0.82	0.18	0.22	0.24	0.34
RandomForest	0.91	0.43	0.36	0.78	0.21	0.89	0.19	0.35	0.30	0.46
RandomTree	0.86	0.32	0.24	0.63	0.08	0.69	0.07	0.23	0.09	0.21
REPTree	0.86	0.44	0.28	0.80	0.15	0.80	0.15	0.33	0.26	0.28
SGD	0.93	0.50	0.30	0.81	**0.44**	0.89	**0.48**	0.59	**0.64**	**0.61**
SimpleLogistic	0.91	0.48	0.36	0.76	0.41	0.89	0.53	**0.65**	0.63	0.59
VotedPerceptron	0.81	0.13	0.33	0.85	0.27	0.83	0.23	0.49	0.47	0.54
ZeroR	0.00	0.00	0.00	0.00	0.00	0.00	0.00	0.00	0.00	0.00
**GA-EoC(avg)**	**0.99**	**0.94**	**0.50**	**0.89**	0.36	**0.92**	0.27	0.62	0.57	0.56
GA-EoC (Stdev)	0.007	0.040	0.009	0.037	0.039	0.056	0.046	0.147	0.085	0.118

We used 10-fold cross validation for the experiments with WBC, PIMA and BUPA datasets. For the experiment of AD-18 and MCI-18, we used Ray-AD-Trn-18 dataset for training but TestSetAD and TestSetMCI as testdata set respectively. We trained the classifier with RMoscato-AD-Trn-5 and tested on TestSetAD and TestSetMCI datasets for the experiment of AD-5 and MCI-5, respectively. For UEAB, IAB and UAB experiments, the GA-EoC was trained on their own training datasets and performances have been measured on respective testing datasets. Same training and testing data manipulation approaches have been used to measure the classification performance of all experiments.

**Table 6:** Classification accuracies achieved by the base classifiers and GA-EoC for all experiments.

Classifier	WBC	PIMA	BUPA	AD-18	MCI-18	AD-5	MCI-5	UAB	IAB	UEAB
BayesNet	97.28	74.35	56.81	89.13	63.83	95.65	63.83	78.00	78.80	81.20
DecisionStump	92.42	71.88	61.74	90.22	57.45	90.22	57.45	80.80	79.60	80.80
DecisionTable	94.13	72.40	59.71	90.22	55.32	90.22	55.32	76.40	74.40	77.60
IBk	95.28	70.18	63.19	94.57	65.96	89.13	57.45	86.80	87.60	86.40
J48	95.14	73.83	67.83	94.57	59.57	90.22	63.83	76.80	78.00	76.40
JRip	95.14	74.61	67.83	79.35	65.96	92.39	55.32	81.20	72.80	76.80
LibSVM	95.71	65.10	59.42	92.39	68.09	93.48	68.09	86.80	86.40	88.80
LMT	95.99	77.47	71.59	88.04	**70.21**	94.57	**74.47**	**89.20**	88.80	87.20
Logistic	96.57	77.21	68.99	85.87	**70.21**	94.57	**74.47**	83.60	85.60	80.00
NaiveBayes	95.99	76.30	53.91	93.48	63.83	95.65	65.96	76.80	76.40	76.40
NaiveBayesUpdateable	95.99	76.30	53.91	93.48	63.83	95.65	65.96	76.80	76.40	76.40
OneR	92.70	70.83	55.94	90.22	57.45	90.22	57.45	76.80	74.40	76.80
PART	94.13	74.48	64.06	90.22	65.96	91.30	59.57	76.40	77.60	78.00
RandomForest	95.99	74.22	68.12	89.13	59.57	94.57	59.57	82.80	81.20	84.40
RandomTree	93.71	69.14	63.48	81.52	53.19	83.70	53.19	75.60	70.00	75.20
REPTree	93.85	75.39	65.51	90.22	57.45	90.22	57.45	77.20	74.00	80.00
SGD	96.71	77.99	66.96	90.22	**70.21**	94.57	72.34	88.00	**89.20**	**87.60**
SimpleLogistic	95.99	77.47	69.28	88.04	**70.21**	94.57	**74.47**	**89.20**	88.80	87.20
VotedPerceptron	90.99	65.36	67.54	92.39	63.83	91.30	61.70	84.00	82.40	84.00
ZeroR	65.52	65.10	57.97	45.65	46.81	45.65	46.81	80.00	80.00	80.00
**GA-EoC (avg)**	**99.43**	**97.43**	**75.72**	**94.66**	67.14	**95.91**	62.98	88.40	86.80	86.80
GA-EoC (Stdev)	0.32	1.71	0.48	1.89	2.24	2.01	2.02	4.34	3.03	3.63

We used 10-fold cross validation for the experiments with WBC, PIMA and BUPA datasets. The classifiers have been trained using Ray-AD-Trn-18 dataset and tested on TestSetAD and TestSetMCI, for the experiment of AD-18 and MCI-18, respectively. We trained the classifiers with RMoscato-AD-Trn-5 and tested on TestSetAD and TestSetMCI datasets for the experiment of AD-5 and MCI-5, respectively. For UEAB, IAB and UAB experiments, classifiers were trained on their own training datasets and performances have been measured on respective testing datasets. Same training and testing data manipulation approaches have been used to measure the classification performance in all experiments.

For the WBC dataset, the average accuracy of GA-EoC is 99.43% (Table 6). The best performing base classifier BayesNet achieved 97.28% classification accuracy on this dataset. The GA-EoC achieved 97.43% classification accuracy in PIMA dataset whereas the closest performing base classifier SimpleLogistic achieved 77.99% accuracy. For the BUPA dataset classification, the proposed method has achieved an average accuracy of 75.72% and LMT, the best performing base classifier, achieved an accuracy of 71.59%. The accuracy gaps between the GA-EoC and the best performing base classifier are 2%, 20% and 4% for WBC, PIMA and BUPA datasets, respectively.

From the results in Tables 6 and 5, it is clear that the proposed GA-EoC method exhibited better performance than any of its constituent base classifiers. Moreover, the standard deviations of the GA-EoC scores (both in terms of MCC and accuracy) over 100-runs for all datasets are very low. For example, for the WBC dataset, the standard deviation in MCC is 0.007 which is less than 1%. As similarly low standard deviation value was observed for the BUPA dataset. However, the GA-EoC converges with 5 different MCCs among 100 repeated runs in the WBC dataset with a standard deviation of 0.040. This demonstrates that the proposed GA-EoC method performs consistently. In conclusion, we observed that for all of the UCI-ML datasets, the proposed method produced better classification performance than its base classifiers and there was no single base classifier that performed consistently on all of these three datasets. These results advocate the effectiveness of the proposed GA based ensemble construction technique.

#### Classification Performances on Alzheimer’s Disease Datasets

Next, we applied GA-EoC for classification of Alzheimer’s disease (AD) and non-Alzheimer’s (NAD) using the aforementioned training and testing datasets. We have compared the classification performance of the proposed GA-EoC with the performance achieved by Ray et al. [44] and Ravetti and Moscato [47] using their respective biomarkers. The feature sets for these datasets were available and class distribution was balanced, therefore, neither the (*α*, *β*) − *k* Feature Set selection method nor the class rebalancing phase was necessary.

**Fig 4:**
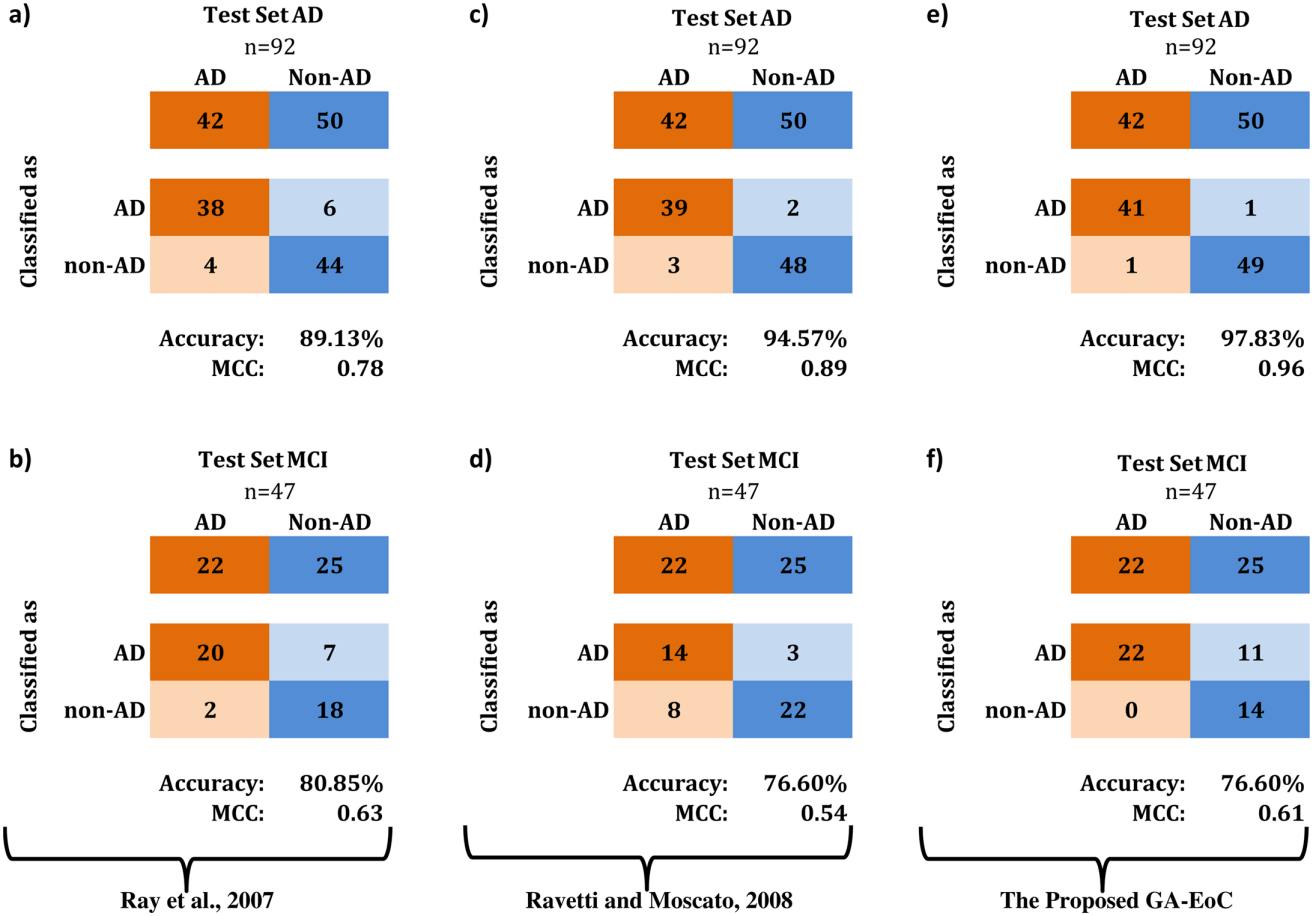
Confusion matrices for comparing the best classification performances using 18-protein biomarker. (a-b) These classification performances are achieved by [Ray et al., 07], (c-d) These classification performances are achieved by [R.Moscato, 08] and (e-f) These classification performances are achieved by the proposed GA-EoC for *TestSetAD* and *TestSetMCI*, respectively.

First, we have compared the classification performances achieved by Ray et al. [44], Ravetti and Moscato [47] and the proposed GA-EoC using 18-protein biomarker. Fig 4 compares the best classification results generated by these three methods using confusion matrices. We also tabulated the average classification performance of these methods in Table 7.

**Table 7:** Average classification performances (in terms of accuracy and MCC) using 18-protein biomarker.

Test Dataset	[Ray et al., 07]		[R.Moscato, 08]		The GA-EoC		Gap	
	*Acc*	*MCC*	*Acc*	*MCC*	*Acc*	*MCC*	*Acc*	*MCC*
Test Set ‘AD’	89.13%	0.78	90.82%	0.82	**94.66%**	**0.89**	3.84%	0.07
Test Set ‘MCI’	**80.85%**	**0.63**	66.19%	0.34	67.14%	0.36	-13.71%	-0.27

For the *TestSetAD* dataset, the PAM classifier used by Ray et al. achieved 89% accuracy with a MCC of 0.78 (Fig 4a). On the other hand, in the experimental setup of Ravetti and Moscato, the best prediction performance for this testing dataset was reported as 95% accuracy with MCC of 0.89 using the IBk classifier (Fig 4c). The best ensemble of classifiers from the proposed GA-EoC outperformed both methods by producing 98% accuracy and a MCC score of 0.96 for the *TestSetAD* dataset (Fig 4e). In terms of average performance, GA-EoC also performed much better than the other two methods (Table 7) in the same dataset.

For the *TestSetMCI*, neither Ravetti-Moscato’s method nor the proposed GA-EoC could exhibit even competitive performance in comparison to the approach of Ray et al (Fig 4b, 4d and 4f). The main reason behind the poor performance of GA-EoC and Ravetti-Moscato’s method on *TestSetMCI* dataset is that the training dataset did not have any samples from the non-AD class (OD and MCI) [47]. If the training dataset contained training samples from the non-AD class, the proposed method could have made more correct predictions for non-AD samples.

However, the proposed method classified Alzheimer’s with 100% positive agreement (Fig 4f) with the follow-up clinical diagnosis, where that rate achieved by Ray et al. was 91% (Fig 4b). Moreover, the average performance of GA-EoC was better than that from Ravetti-Moscato’s method in both scales (Table 7).

Next, the 5-protein biomarker discovered by Ravetti and Moscato [47] was used as features for classifying Alzheimer’s disease dataset. The average performance of GA-EoC in accuracy and MCC scale is compared with that of Ravetti-Moscato’s method in Table 8. We can observe that the GA-EoC produced a better accuracy (3% more classification accuracy than that reported in [47]) and MCC (0.06 more than that achieved in [47]) for prediction in the *TestSetAD* dataset. On the prediction of *TestSetMCI*, the proposed method performed worse than Ravetti-Moscato’s method (1.6% less in accuracy and 0.03 less in MCC score).

**Table 8:** Average classification performances (in terms of accuracy and MCC) using 5-protein biomarker.

Test Dataset	[R.Moscato, 08]		The GA-EoC		Gap	
	*Acc*	*MCC*	*Acc*	*MCC*	*Acc*	*MCC*
Test Set ‘AD’	92.90%	0.86	**95.91%**	**0.92**	3.01%	0.06
Test Set ‘MCI’	**64.55%**	**0.3**	62.98%	0.27	-1.57%	-0.03

**Fig 5:**
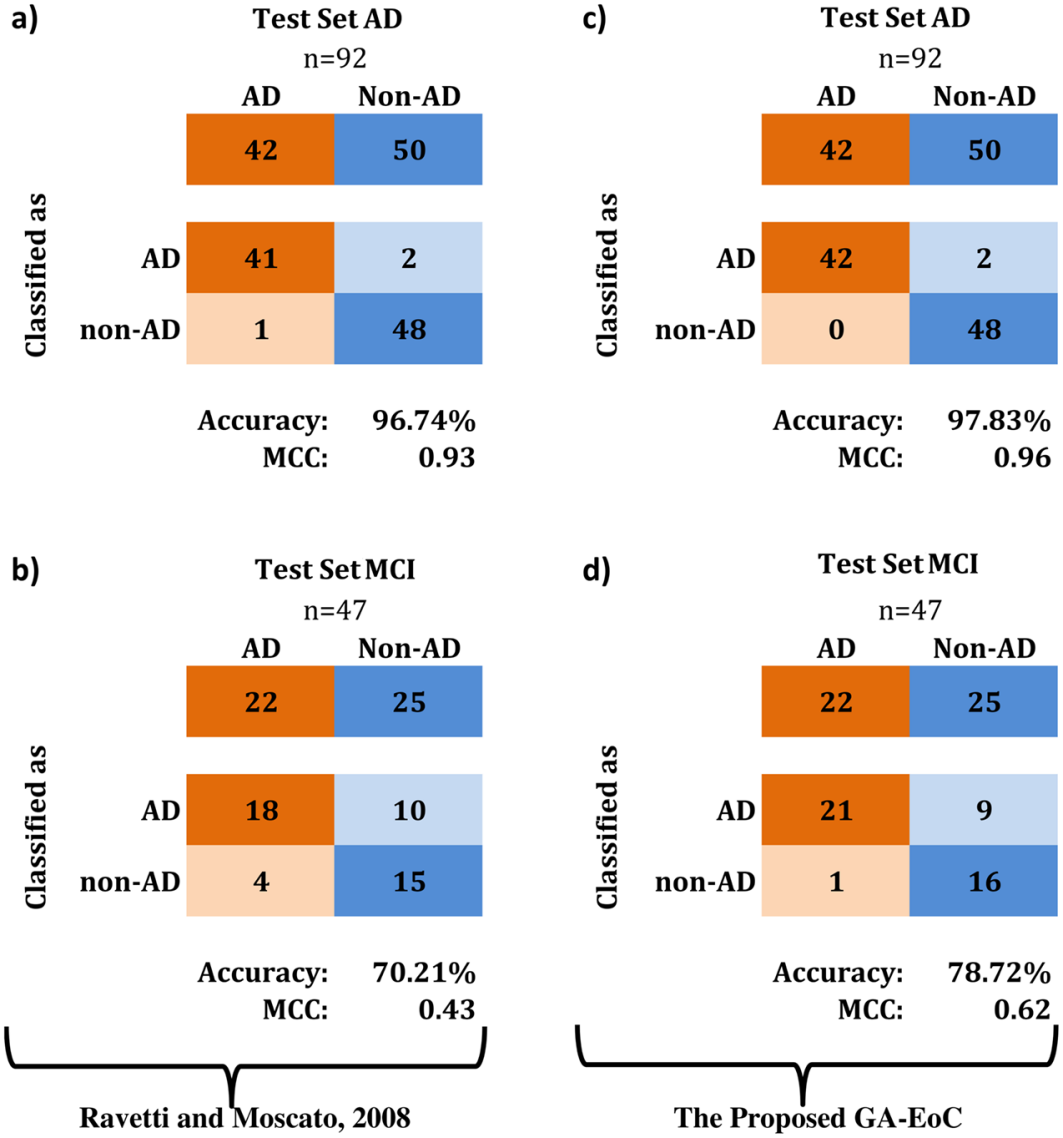
Best classification performances by the state of art method vs. the proposed method with the 5-protein biomarker. The comparison of best classification performances using the 5-protein biomarker (RavettiMoscato-AD-Trn-5) as training dataset and *TestSetAD* and *TestSetMCI* as test datasets. (a-b) Classification performances achieved by [R.Moscato, 08], (c-d) Classification performances achieved by GA-EoC for the *TestSetAD* and *TestSetMCI*, respectively.

For better understanding the detailed performances, we have showed the confusion matrices of the best performance achieved by Ravetti-Moscato’s method and GA-EoC in Fig 5. The best classification performance achieved by GA-EoC using the 5-protein biomarker produced 98% accuracy with 0.96 MCC for the *TestSetAD* (Fig 5c) and 79% accuracy with 0.62 MCC score for the *TestSetMCI* datasets (Fig 5d). The best ensemble combination of GA-EoC produced better generalisation performance for all of the datasets tested by Ravetti and Moscato [47]. Only for the MCI datasets, the average performance of GA-EoC was slightly poorer compared to the other method. The reason why the GA-EoC failed to perform well in MCI datasets classification is explained in the previous section.

Finally, if we compare the performance of GA-EoC with that of the base classifiers (Tables 6 and 5), then it is found that GA-EoC consistently performed at least as well as its base classifiers in classifying *TestSetAD* using both 18 and 5 biomarkers. But in case of *TestSetMCI* the performance of GA-EoC was found to be poor compared to some of its base classifiers. As we have explained, this poor performance was due to the biased nature of the dataset.

#### Classification Performances on Face Recognition Dataset

We obtained three configured datasets (named UAB, IAB and UEAB datasets) from the *PubFig*05 dataset after class rebalancing and applying entropy filtering and (*α*, *β*) − *k* Feature Set selection methods as explained in the Datasets section. In other words, all the preprocessing phases were used for these datasets before applying the GA-EoC algorithm. Each of these configured datasets is comprised of five binary-class datasets. The generalisation performances achieved by GA-EoC on these datasets are very close to the best base classifiers’ performances. The accuracy gap between the best base classifier and the average accuracy of proposed method is less than 2.5% (Table 5). In terms of MCC, this gap lies below 0.08 (Table 6).

We trained the proposed GA-EoC and three other ensemble methods namely Bagging, AdaBoostM1 and Random Forest on these datasets and compared their classification performances on testing datasets. The classification performance of these algorithms were compared on the scale of precision, accuracy, F-Measure and MCC.

**Fig 6:**
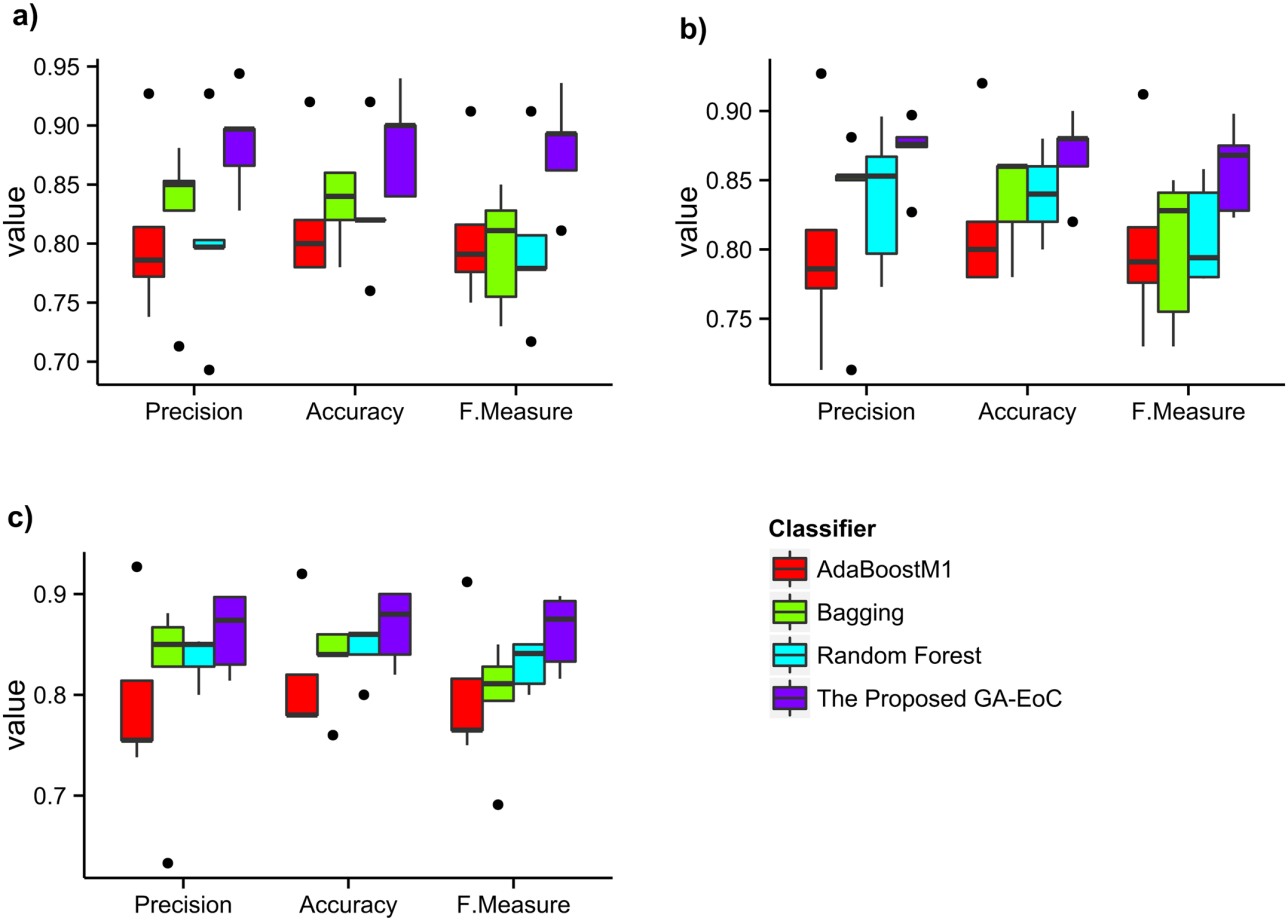
Classification performances of GA-EoC and other ensemble of classifiers on PubFig05 datasets. The classification performances of AdaBoostM1, Bagging, Random Forest and GA-EoC are compared in terms of Precision, Accuracy and F-Measure scores for (a) UAB datasets, (b) IAB datasets and (c) UEAB datasets.

- UAB datasets: We tabulated the average classification performance of the proposed method and three other ensemble methods for the five binary-class datasets in UAB settings in Table 9. GA-EoC achieved average MCC of 0.623 and average accuracy of 88%. The closest performing ensemble method, AdaBoostM1, achieved an average MCC of 0.387 with accuracy of 82%. Fig 6a illustrates the performances of different EoCs using box-plots in terms of Precision, Accuracy and F-Measures. We can observe that the proposed GA-EoC method clearly outperformed other ensembles of classifiers in all measures.
- IAB datasets: The average classification performances achieved by participating ensembles are given in Table 10 for IAB datasets. Here also, GA-EoC outperformed all ensemble methods used in the experiment by achieving 0.565 average MCC with average accuracy of 86.80%. The closest ensemble method, the Random Forest, produced 0.414 MCC with 84% accuracy for IAB datasets on an average. From Fig 6b, that compares the classification performances of different ensembles in different measures, we can see that GA-EoC outperformed other ensemble methods in all reported measures.
- UEAB datasets: Finally, we performed experiments on the UEAB datasets. The GA-EoC achieved 0.564 MCC with accuracy of 87% (Table 11) which is very similar to the results on IAB datasets. The proposed method outperformed the other ensemble methods as well for the UEAB setup of datasets. In this case, it used less than one third of the features that were used in the IAB dataset, but achieved almost the same MCC, accuracy and f-measure scores. The overall performances of GA-EoC and other ensembles are illustrated in the box-plot of Fig 6c in different measures. It is clear from the figure that once again GA-EoC outperformed other ensemble classifiers in this dataset in terms of all reported measures.

**Table 9:** Average classification performances on UAB setup.

Classifier	Precision	Accuracy	F-Measure	MCC
Bagging	0.825	83.20%	0.795	0.36
AdaBoostM1	0.807	82.00%	0.809	0.387
Random Forest	0.803	82.80%	0.799	0.348
GA-EoC	**0.886**	**88.40%**	**0.879**	**0.623**

**Table 10:** Average classification performances on IAB setup.

Classifier	Precision	Accuracy	F-Measure	MCC
Bagging	0.83	83.60%	0.801	0.379
AdaBoostM1	0.802	82.00%	0.805	0.370
Random Forest	0.837	84.00%	0.810	0.414
GA-EoC	**0.871**	**86.80%**	**0.858**	**0.565**

**Table 11:** Average classification performances on UEAB setup.

Classifier	Precision	Accuracy	F-Measure	MCC
Bagging	0.812	83.20%	0.795	0.347
AdaBoostM1	0.798	81.60%	0.802	0.357
Random Forest	0.836	84.40%	0.830	0.464
GA-EoC	**0.862**	**86.80%**	**0.863**	**0.564**

Since MCC is the representative performance measure in this work, we analysed the MCC scores achieved by GA-EoC and other ensembles for the face recognition problem datasets separately in Fig 6. Among the three dataset configurations, GA-EoC performed best in UAB setup. The 25^*th*^ percentile of MCC score achieved by the GA-EoC is nearly 0.60. Whereas, the Bagging classifier’s 50^*th*^ percentile MCC score is below 0.40, which is the closest performing classifier for this configuration. In IAB configuration, the 50^*th*^ percentile of MCC is nearly 0.60 for GA-EoC. The closest performing ensemble classifiers, Bagging and Random Forest’s 50^*th*^ percentile MCC scores are below 0.50 and 0.40 respectively, which shows that GA-EoC performed far better than other ensembles of classifiers in this dataset also. The 50^*th*^ percentile MCC scores for the GA-EoC are above 0.60 for the UEAB configuration. In comparison to that, the rest of the classifier’s 75^*th*^ percentile MCC scores are below 0.50 in MCC scale. The confusion matrices depicting the outcomes of classification by GA-EoC have been included in the supporting information S1 File. It is also notable that the UEAB configured datasets contain least number of features (average number of features was 1700 per binary-dataset) among these three configurations. However, the classification performance achieved by GA-EoC using these features are comparable to previous two tests. It has been observed from the result, a compact and good set of features selected by the (*α*, *β*) − *k* feature selection method helped GA-EoC to achieve good generalisation performance. Nevertheless, the other ensembles, using the same set of features, could not generate similar performance. These experimental outcomes advocate for the effectiveness of the (*α*, *β*) − *k* feature selection method and qualify it as a candidate for the dimensionality reduction techniques to be used in GA-EoC.

The performance comparison presented in Table 5 shows that there is no single classifier that achieves the best accuracy for all experiments done with different types of datasets. Among the 20 base classifiers, LMT and SimpleLogistic were able to achieve the best accuracy for 4 experiments which include both MCI experiments. The SGD and Logistic were able to produce the best classification accuracy for 3 (one MCI experiment included) and 2 (both MCI experiments included) experiments, respectively. GA-EoC outperformed the best accuracies of all base classifiers for 5 experiments. Moreover, the average accuracies of GA-EoC are also close (3% less than the best accuracy for other experiments) to the best classification accuracies for other experiments.

**Fig 7:**
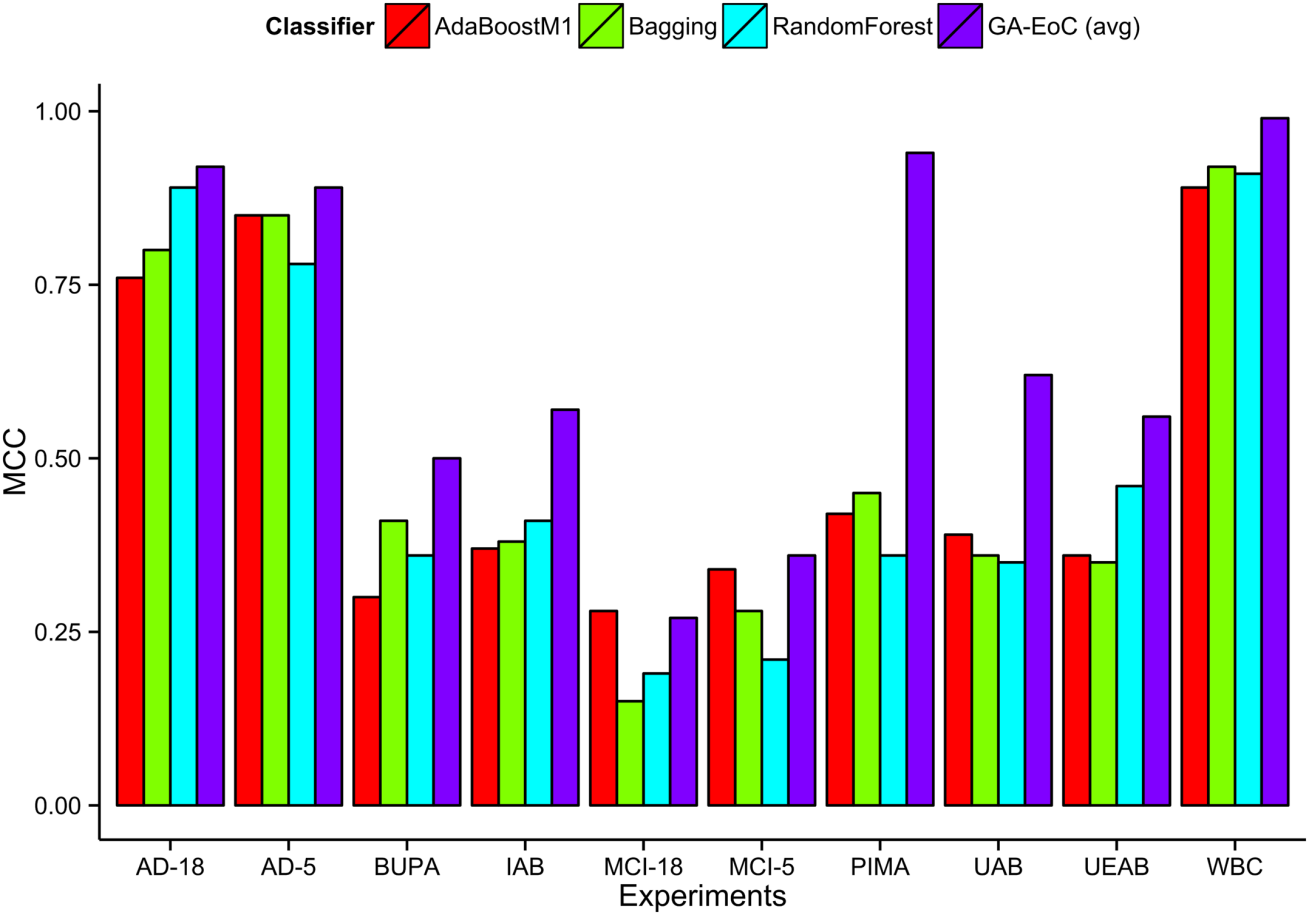
Comparison of MCC scores achieved by GA-EoC and other ensemble of classifiers (AdaBoostM1, Bagging and Boosting) for all experiments.

If we consider the MCC scores presented in Table 6, only SGD classifier was able to achieve the best MCC for 4 experiments, which include two MCI experiments. For the other experiments, the best MCC is achieved by each of the four different base classifiers in one experiment. The average MCC value of GA-EoC outperformed the best base classifier’s MCC value for 5 experiments. Moreover, for the other 3 experiments the average MCC scores of GA-EoC are close to the best performing base classifier. Therefore, summarising all the results from Tables 6 and 5, it can be concluded that the average performance of GA-EoC, over a variety of classes of datasets, is better than any single base classifier. This is further illustrated in Fig 7.

**Fig 8:**
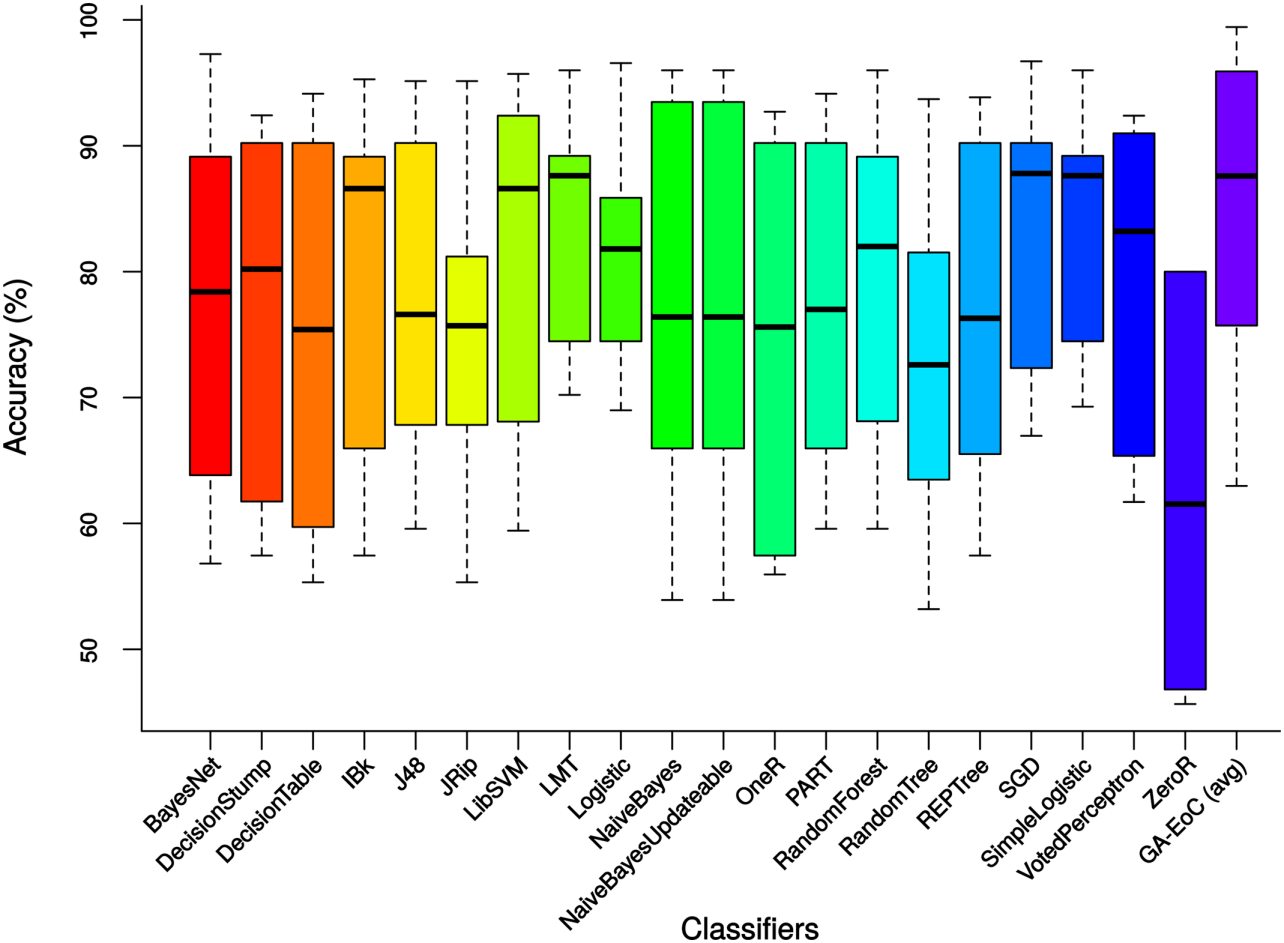
The accuracies of base classifiers and average accuracies of GA-EoC over all experiments.

**Fig 9:**
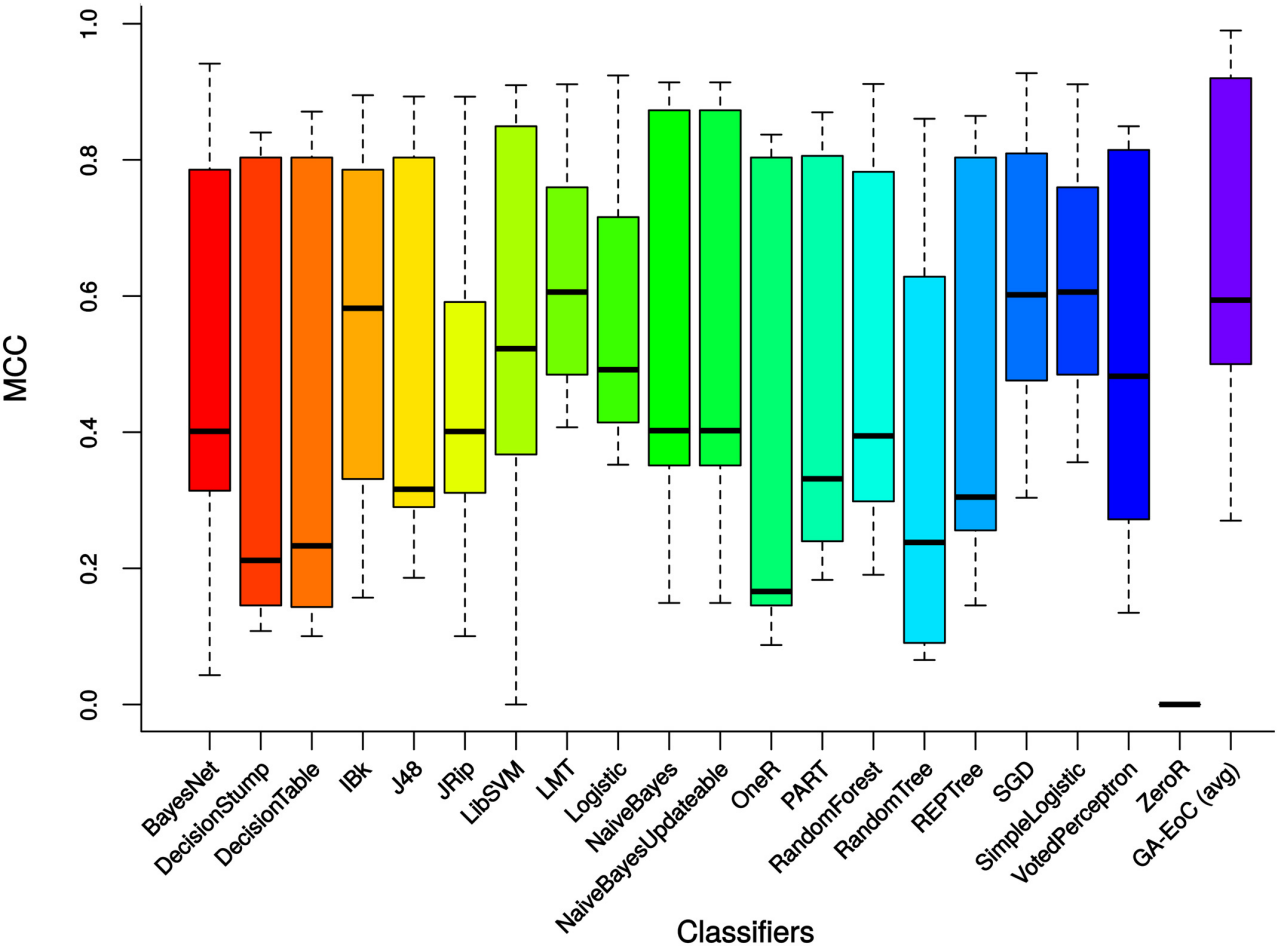
The MCC scores of base classifiers and the average MCC scores of GA-EoC over all experiments.

The box-plots in Fig 8 compare the average classification accuracy achieved by the proposed GA-EoC and the base classifiers over all experiments. From the box plot, it is clear that the median or 50^*th*^ percentile accuracy of GA-EoC is similar to accuracies achieved by LMT, SGD and SimpleLogistic classifiers. But the 75^*th*^ percentile accuracy of the GA-EoC is higher than all of them. In terms of accuracy, GA-EoC performed better than the base classifiers considering all test cases. The box-plot in Fig 9 shows the classification performances of base classifiers and the average performance of the GA-EoC for all datasets using the MCC scale. The median of the MCCs achieved by the GA-EoC is similar to MCCs of LMT, SGD and SimpleLogistic classifiers. However, the 75^*th*^ percentile MCC score of GA-EoC is once again found to be better than any of the base classifier considering all experiments. These results show the robustness of GA-EoC as a single method compared to other base classifiers.

Next, we investigate the types of base classifiers selected in GA-EoC ensemble pool in different experimental runs. In Table 12 we list the common base classifiers included in the ensembles constructed by GA-EoC for all experiments. The proposed method selected only 7 different combinations of base classifiers over 100 runs in PIMA dataset experiment. We found that DecisionStump, IBk, RandomForest, RandomTree classifiers are common among all ensembles. None of these base classifier’s MCC score was over 0.45 but their ensemble produced an average MCC score of 0.94. Moreover, in terms of MCC, the SGD classifier was the best base classifier for PIMA dataset, but it has not appeared in any of the best ensembles selected by the GA-EoC. Analysis of the population revealed that although SGD appeared in individuals in early generations, it did not survive due to low fitness in the genetic algorithm. For an example, 01010010000001101000 is one of the individuals that contains the SGD classifier and produces MCC of 0.9153 which is lower than the optimised fitness value. From this observation, it becomes clear that ensembles created with best base classifiers do not always produce a better MCC, but a combination of diverse base classifiers could produce better outcomes. In other datasets, we also found that there were only a few different ensemble combinations in which GA-EoC converged over repeated runs. This observation supports our claim that GA-EoC is consistent in generating ensembles.

**Table 12:** The number of different ensembles (with common base classifiers in them) constructed by GA-EoC over repeated experimental runs.

Training Dataset	Common Base Classifier	#Different Ensemble
BUPA	JRip, RandomTree, SGD	6
PIMA	DecisionStump, IBk, RandomForest, RandomTree	7
WBC	JRip, LibSVM, SGD	8
RMoscato-AD-Trn-5	JRip, LibSVM	6
Ray-AD-Trn-18	JRip	20
IAB	IBk, JRip, Logistic, PART, SGD, VotedPerceptron	5
UAB	IBk, Logistic, RandomTree	5
UEAB	IBk, Logistic, RandomTree	5

Finally, we compared GA-EoC with other common ensemble of classifiers (Bagging, AdaBoostM1 and Random Forest), over all experimental datasets. We used the default parameter settings for those ensembles of classifier algorithms available in WEKA framework. The classification performances achieved by other ensemble methods and GA-EoC are shown in Table 13. The average accuracy of GA-EoC is better than those classifiers for all test cases. In terms of MCC score, AdaBoostM1 marginally outperformed GA-EoC only for the MCI-5 experiment. In other experiments, GA-EoC have achieved better average MCC score than other ensembles. Based on these results we claim that, GA-EoC is a better choice than many other ensembles of classifiers like Bagging, AdaBoostM1 and Random Forest for imbalanced-class datasets.

**Table 13:** Classification performances of common ensemble of classifiers vs GA-EoC for all experiments.

Experiments	AdaBoostM1		Bagging		RandomForest		GA-EoC (avg)	
	*Acc*	*MCC*	*Acc*	*MCC*	*Acc*	*MCC*	*Acc*	*MCC*
WBC	95.14	0.89	96.28	0.92	95.99	0.91	**99.43**	**0.99**
PIMA	74.35	0.42	75.78	0.45	72.14	0.36	**97.43**	**0.94**
BUPA	66.96	0.30	71.88	0.41	68.12	0.36	**75.72**	**0.50**
AD-18	92.39	0.85	92.39	0.85	89.13	0.78	**94.66**	**0.89**
MCI-18	65.96	0.34	63.83	0.28	59.57	0.21	**67.14**	**0.36**
AD-5	86.96	0.76	90.22	0.80	94.57	0.89	**95.91**	**0.92**
MCI-5	59.57	**0.28**	57.45	0.15	59.57	0.19	**62.98**	0.27
UAB	82.00	0.39	83.20	0.36	82.80	0.35	**88.40**	**0.62**
IAB	82.00	0.37	83.60	0.38	84.00	0.41	**86.80**	**0.57**
UEAB	81.60	0.36	83.20	0.35	84.40	0.46	**86.80**	**0.56**

## Conclusion

This work presents a genetic algorithm based search method, named GA-EoC, for constructing heterogeneous ensembles of classifiers. As the number of base classifiers increases, the number of possible ensembles that can be created rises exponentially. Since an exhaustive search for constructing the best ensemble is not feasible, we propose a genetic algorithm for searching the best combination of base classifiers for constructing the ensemble. GA-EoC employs the majority voting technique for combining the base classifier’s decisions in a single final decision.

Imbalanced class distributions in many real-world datasets has become a significant challenge for a classifier’s performance. In case of imbalanced datasets, the GA-EoC, as a preprocessing step, creates several balanced datasets depending on the imbalance ratio between two classes of the imbalanced dataset. We have applied the (*α*, *β*) − *k* Feature Set selection method for the datasets where no feature set was given *a priori* for classification. GA-EoC generates the models of base classifiers using 10-fold cross validation on these balanced training datasets and then reuses them while evaluating different combinations of heterogeneous ensemble.

The performance of GA-EoC has been evaluated using various datasets selected from UCI-ML repository and other sources. These experimental results suggest that the ensembles constructed by GA-EoC are better than a single base classifier in general. Moreover, the ensembles constructed by GA-EoC were found to be better than those constructed by many established ensemble constructed methods.

In this work GA-EoC has been studied in a simple setting which can be improved and extended in many ways. For example, better classification accuracy can be achieved by fine-tuning the parameters (i.e. rule weights, membership functions, etc.) of base classifiers or utilising the other fusion approaches [14]. In the current implementation of the GA-EoC, a single objective GA is used to optimise the MCC score of the ensemble. The work can be extended by using multi-objective GAs to handle more challenging and complex classification problems. Incorporation of such advanced and efficient components can improve the generalisation performance of GA-EoC and be more capable of handling the class-imbalanced and dimensionality challenges in modern datasets.

## Supporting Information

S1 File Supporting information S1 File contains details about pre-processing applied on *PubFig*05 datasets, confusion matrices for experiments on *PubFig*05 datasets and base classifiers performances for all experiments. The step by step outcomes of data pre-processing applied on the *PubFig* − 05 dataset for UAB configuration (Table A), IAB configuration (Table B) and UEAB configurations (Table C). Base classifier’s performances (the MCC, Accuracy, F-Measure and Precision scores) are shown for datasets WBC (Table D), BUPA (Table E), PIMA (Table F), AD using 5-protein biomarker (Table G), MCI using 5-protein biomarker (Table H), AD using 18-protein biomarker (Table I), MCI using 18-protein biomarker (Table J), UAB (Table K), IAB (Table L) and UEAB (Table M). Confusion Matrices for the GA-EoC are shown for UAB (Fig A), IAB (Fig B) and UEAB (Fig C). Comparison of MCC scores achieved by the GA-EoC and other ensemble of the classifiers are shown in Fig D.
(PDF)

S2 File Readme file for the GA-EoC program. This supporting information file describes about available features, system requirements and how to use the GA-EoC program.
(PDF)
