# Supplementary material for: Heterogeneous Ensemble Combination Search Using Genetic Algorithm for Class Imbalanced Data Classification
Source: PLoS One. 2016 Jan 14;11(1):e0146116. doi: 10.1371/journal.pone.0146116 (PMC4713117; doi:10.1371/journal.pone.0146116)
Supplement: S1 File — The step by step outcomes of data pre-processing applied on the PubFig − 05 dataset for UAB configuration (Table A), IAB configuration (Table B) and UEAB configurations (Table C). Base classifier’s performances (the MCC, Accuracy, F-Measure and Precision scores) are shown for datasets WBC (Table D), BUPA (Table E), PIMA (Table F), AD using 5-protein biomarker (Table G), MCI using 5-protein biomarker (Table H), AD using 18-protein biomarker (Table I), MCI using 18-protein biomarker (Table J), UAB (Table K), IAB (Table L) and UEAB (Table M). Confusion Matrices for the GA-EoC are shown for UAB (Fig A), IAB (Fig B) and UEAB (Fig C). Comparison of MCC scores achieved by the GA-EoC and other ensemble of the classifiers are shown in Fig D. (PDF) [file pone.0146116.s001.pdf]

Supporting Information File S1

December 19, 2015

The supporting information file contains more experimental results which are not included in the paper. It shows the step by step feature selection outcomes for UAB, IAB and UEAB configured datasets. It also contains detail classification performances (using MCC, accuracy, F-Measure and precision scores) for all experiments and confusion matrices of $PubFig05$ datasets.

List of Tables

A Details about the feature selection by (α, β)-k Feature Set method for the *UAB* configuration. 3
B Details about the feature selection by (α, β)-k Feature Set method for the *IAB* configuration. 4
C Details about the feature selection by (α, β)-k Feature Set method for the *UEAB* configuration. 5
D Classification performances of base classifiers for the WBC dataset. 6
E Classification performances of base classifiers for the BUPA dataset. 7
F Classification performances of base classifiers for the PIMA dataset. 8
G Classification performances of base classifiers for the AD dataset using the 5-protein biomarker. 9
H Classification performances of base classifiers for the MCI dataset using the 5-protein biomarker. 10
I Classification performances of base classifiers for the AD dataset using the 18-protein biomarker. 11
J Classification performances of base classifiers for the MCI dataset using the 18-protein biomarker. 12
K Classification performances of base classifiers for the UAB datasets. 13
L Classification performances of base classifiers for the IAB datasets. 14
M Classification performances of base classifiers for the UEAB datasets. 15

List of Figures

A Confusion matrices to show the classification performances of the proposed GA-EoC for the *UAB* configuration of "one-vs-all" setup. 6

B Confusion matrices to show the classification performances of the proposed GA-EoC for the *IAB* configuration of “one-vs-all” setup. 7
C Confusion matrices to show the classification performances of the proposed GA-EoC for the *UEAB* configuration of “one-vs-all” setup. 8
D Comparison of MCCs achieved by the GA-EoC and state-of-the-art ensemble of classifiers for different configuration of PubFig-05 datasets. 9

Table A: This table represents the number of features selected by $(\alpha, \beta) - k$ feature set method for the UAB setup. In this data pre-processing, we applied entropy filtering on the whole multi-class dataset at the beginning and converted it into five (05) binary-class datasets. Then, we separated each of them into four balanced binary-class datasets using random sampling method. Afterwards, we applied $(\alpha, \beta) - k$ feature set method (ABK) on each of the balanced binary class-datasets and we took the **union** of selected features for each binary-class datasets. Finally, we applied the $(\alpha, \beta) - k$ feature set selection method on each of the binary-class datasets and get a set of features.

**# Features**	**# Features : Entropy**	**Binary-Class**	**Balanced Binary**	**#Feature : ABK**	**# ∪ Features**	**#Feature : ABK**
25600	4878	Cls 0 vs all	Cls 0 0	4719	4828	4656
			Cls 0 1	4642		
			Cls 0 2	4725		
			Cls 0 3	4750		
		Cls 1 vs all	Cls 1 0	4706	4855	4702
			Cls 1 1	4800		
			Cls 1 2	4736		
			Cls 1 3	4802		
		Cls 2 vs all	Cls 2 0	4743	4835	4712
			Cls 2 1	4687		
			Cls 2 2	4704		
			Cls 2 3	4762		
		Cls 3 vs all	Cls 3 0	4743	4855	4678
			Cls 3 1	4629		
			Cls 3 2	4799		
			Cls 3 3	4799		
		Cls 4 vs all	Cls 4 0	4713	4834	4738
			Cls 4 1	4735		
			Cls 4 2	4774		
			Cls 4 3	4719		

Table B: This table represents the number of features selected by $(\alpha, \beta) - k$ feature set method for the ***Intersection of ABK (Balanced Binary)*** **datasets (IAB)** setup. In this data pre-processing, we applied entropy filtering on the whole multi-class dataset at the beginning and converted it into five (05) binary-class datasets. Then, we separated each of them into four balanced binary-class datasets using random sampling method. Afterwards, we applied $(\alpha, \beta) - k$ feature set method on each of the balanced binary class-datasets and we took the **intersection** of selected features for each binary-class datasets. Finally, we applied the $(\alpha, \beta) - k$ feature set selection method (ABK) on each of the binary-class datasets and get a set of features.

# Features	# Features : Entropy	Binary-Class	Balanced Binary	#Feature : ABK	# ∩ Features	#Feature : ABK
25600	4878	Cls 0 vs all	Cls 0 0	4719	4544	4495
			Cls 0 1	4642		
			Cls 0 2	4725		
			Cls 0 3	4750		
		Cls 1 vs all	Cls 1 0	4706	4616	4598
			Cls 1 1	4800		
			Cls 1 2	4736		
			Cls 1 3	4802		
		Cls 2 vs all	Cls 2 0	4743	4585	4563
			Cls 2 1	4687		
			Cls 2 2	4704		
			Cls 2 3	4762		
		Cls 3 vs all	Cls 3 0	4743	4561	4501
			Cls 3 1	4629		
			Cls 3 2	4799		
			Cls 3 3	4799		
		Cls 4 vs all	Cls 4 0	4713	4602	
			Cls 4 1	4735		
			Cls 4 2	4774		
			Cls 4 3	4719		

Table C: This table represents the number of features selected by $(\alpha, \beta) - k$ feature set method for the ***Union of Entf ABK (Balanced Binary)* datasets (UEAB)** setup. In this data pre-processing, we applied entropy filtering on the whole multi-class dataset at the beginning and converted it into five (05) binary-class datasets. Then, we separated each of them into four balanced binary-class datasets using random sampling method and applied entropy filtering on each of them. Afterwards, we applied $(\alpha, \beta) - k$ feature set method (ABK) on each of the entropy filtered balanced binary class-datasets and we took the **union** of selected features for each binary-class datasets. Finally, we applied the ABK on each of the binary-class datasets and get a set of features.

# Features	# Features : Entropy	Binary-Class	Balanced Binary	# Features : Entropy	# Featutres : ABK	# ∪ Feature
25600	4878	Cls 0 vs all	Cls 0 0	493	285	798
			Cls 0 1	634	294	
			Cls 0 2	594	384	
			Cls 0 3	609	333	
		Cls 1 vs all	Cls 1 0	1313	851	1554
			Cls 1 1	1192	522	
			Cls 1 2	1368	858	
			Cls 1 3	1345	684	
		Cls 2 vs all	Cls 2 0	1726	1278	2273
			Cls 2 1	1458	1068	
			Cls 2 2	1789	1263	
			Cls 2 3	1810	1329	
		Cls 3 vs all	Cls 3 0	2334	1278	2821
			Cls 3 1	2435	1743	
			Cls 3 2	2297	1763	
			Cls 3 3	2106	1760	
		Cls 4 vs all	Cls 4 0	1018	592	1081
			Cls 4 1	694	443	
			Cls 4 2	748	386	
			Cls 4 3	583	386	

Table D: Classification performances of base classifiers for the WBC dataset.

Classifier	MCC	Accuracy (%)	FMeasure	Precision
BayesNet	**0.941**	**97.28**	**0.979**	**0.993**
DecisionStump	0.840	92.42	0.940	0.972
DecisionTable	0.871	94.13	0.955	0.960
IBk	0.895	95.28	0.964	0.957
J48	0.893	95.14	0.963	0.969
JRip	0.893	95.14	0.963	0.967
LibSVM	0.910	95.72	0.966	0.993
LMT	0.911	95.99	0.970	0.965
Logistic	0.924	96.57	0.974	0.974
NaiveBayes	0.914	95.99	0.969	0.986
NaiveBayesUpdateable	0.914	95.99	0.969	0.986
OneR	0.837	92.70	0.946	0.923
PART	0.870	94.13	0.955	0.952
RandomForest	0.912	95.99	0.969	0.971
RandomTree	0.860	93.71	0.952	0.950
REPTree	0.865	93.85	0.953	0.958
SGD	0.927	96.71	0.975	0.978
SimpleLogistic	0.911	95.99	0.970	0.965
VotedPerceptron	0.815	90.99	0.928	0.974
ZeroR	0.000	65.52	0.792	0.655

Figure A: Confusion matrices to show the classification performances of the proposed GA-EoC for the *UAB* configuration of "one-vs-all" setup.

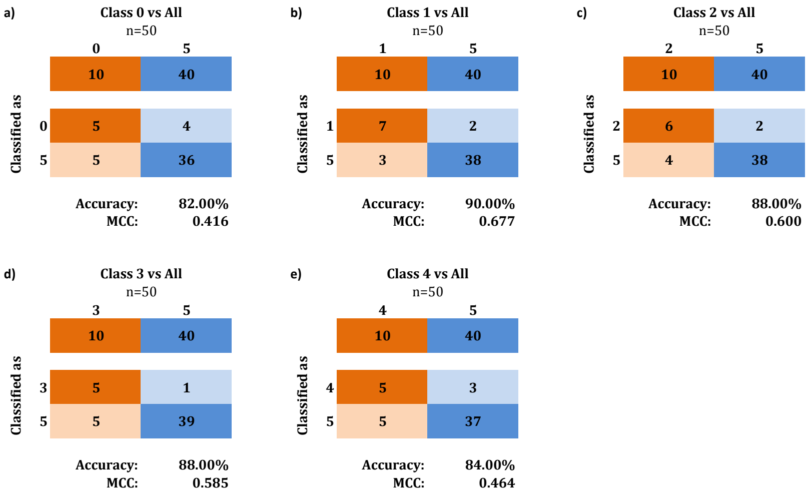

Table E: Classification performances of base classifiers for the BUPA dataset.

Classifier	MCC	Accuracy (%)	FMeasure	Precision
BayesNet	0.043	56.81	0.273	0.467
DecisionStump	0.201	61.74	0.511	0.552
DecisionTable	0.143	59.71	0.437	0.529
IBk	0.241	63.19	0.554	0.564
J48	0.328	67.83	0.581	0.642
JRip	0.325	67.83	0.565	0.655
LibSVM	0.127	59.42	0.079	**0.857**
LMT	**0.407**	**71.59**	0.626	0.701
Logistic	0.352	68.99	0.596	0.658
NaiveBayes	0.149	53.91	0.583	0.470
NaiveBayesUpdateable	0.149	53.91	0.583	0.470
OneR	0.087	55.94	0.457	0.474
PART	0.261	64.06	0.569	0.573
RandomForest	0.355	68.12	**0.638**	0.610
RandomTree	0.243	63.48	0.547	0.571
REPTree	0.277	65.51	0.548	0.610
SGD	0.304	66.96	0.533	0.657
SimpleLogistic	0.356	69.28	0.579	0.682
VotedPerceptron	0.333	67.54	0.446	0.789
ZeroR	0.000	57.97	0.000	0.000

Figure B: Confusion matrices to show the classification performances of the proposed GA-EoC for the *IAB* configuration of "one-vs-all" setup.

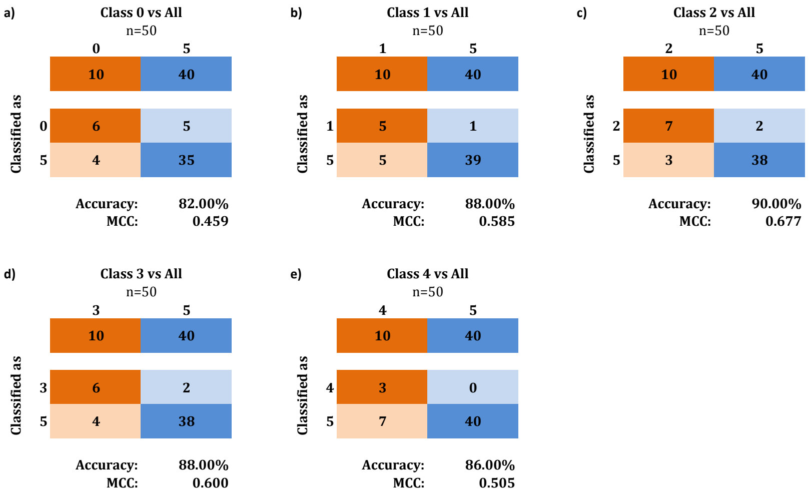

Table F: Classification performances of base classifiers for the PIMA dataset.

Classifier	MCC	Accuracy (%)	FMeasure	Precision
BayesNet	0.429	74.35	0.623	0.639
DecisionStump	0.375	71.88	0.588	0.602
DecisionTable	0.377	72.40	0.579	0.619
IBk	0.331	70.18	0.554	0.580
J48	0.417	73.83	0.614	0.632
JRip	0.434	74.61	0.626	0.644
LibSVM	0.000	65.10	0.000	0.000
LMT	0.485	77.47	0.634	0.732
Logistic	0.480	77.21	0.636	0.718
NaiveBayes	0.468	76.30	**0.643**	0.678
NaiveBayesUpdateable	0.468	76.30	**0.643**	0.678
OneR	0.329	70.83	0.531	0.605
PART	0.435	74.48	0.629	0.638
RandomForest	0.434	74.22	0.632	0.630
RandomTree	0.318	69.14	0.554	0.559
REPTree	0.444	75.39	0.623	0.670
SGD	**0.497**	**77.99**	0.641	**0.744**
SimpleLogistic	0.485	77.47	0.634	0.732
VotedPerceptron	0.135	65.36	0.289	0.509
ZeroR	0.000	65.10	0.000	0.000

Figure C: Confusion matrices to show the classification performances of the proposed GA-EoC for the *UEAB* configuration of "one-vs-all" setup.

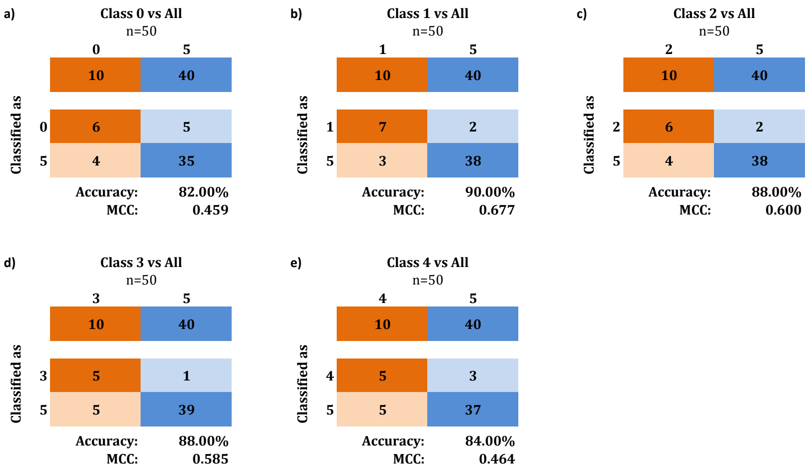

Table G: Classification performances of base classifiers for the AD dataset using the 5-protein biomarker.

Classifier	MCC	Accuracy (%)	FMeasure	Precision
BayesNet	**0.914**	**95.65**	**0.953**	0.932
DecisionStump	0.803	90.22	0.889	0.923
DecisionTable	0.803	90.22	0.889	0.923
IBk	0.786	89.13	0.886	0.848
J48	0.803	90.22	0.894	0.884
JRip	0.848	92.39	0.914	0.949
LibSVM	0.870	93.48	0.930	0.909
LMT	0.893	94.56	0.943	0.911
Logistic	0.893	94.56	0.943	0.911
NaiveBayes	**0.914**	**95.65**	**0.953**	0.932
NaiveBayesUpdateable	**0.914**	**95.65**	**0.953**	0.932
OneR	0.803	90.22	0.889	0.923
PART	0.825	91.30	0.902	0.925
RandomForest	0.890	94.56	0.940	**0.951**
RandomTree	0.690	83.70	0.839	0.765
REPTree	0.803	90.22	0.889	0.923
SGD	0.893	94.56	0.943	0.911
SimpleLogistic	0.893	94.56	0.943	0.911
VotedPerceptron	0.827	91.30	0.900	0.947
ZeroR	0.000	45.65	0.627	0.457

Figure D: Comparison of MCCs achieved by the GA-EoC and state-of-the-art ensemble of classifiers for different configuration of PubFig-05 datasets.

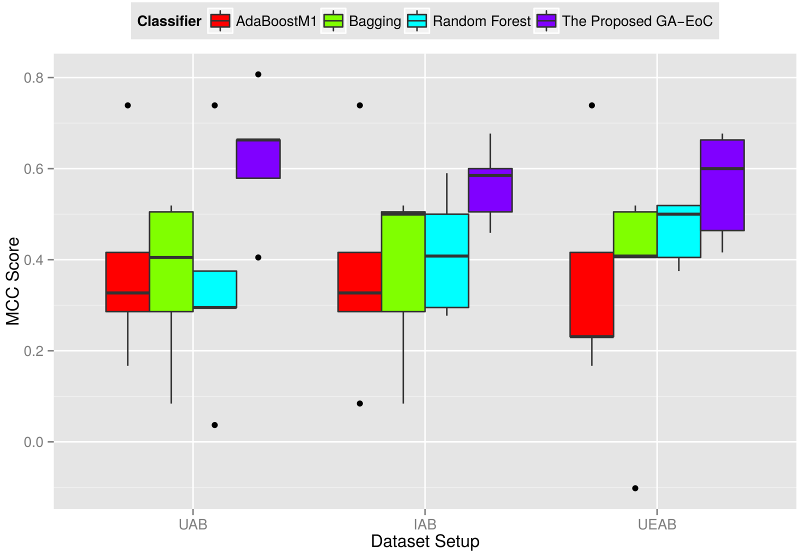

Table H: Classification performances of base classifiers for the MCI dataset using the 5-protein biomarker.

**Classifier**	**MCC**	**Accuracy (%)**	**FMeasure**	**Precision**
BayesNet	0.282	63.83	0.638	0.600
DecisionStump	0.145	57.45	0.545	0.545
DecisionTable	0.100	55.32	0.512	0.524
IBk	0.157	57.45	0.583	0.538
J48	0.272	63.83	0.605	0.619
JRip	0.100	55.32	0.512	0.524
LibSVM	0.367	68.08	0.681	0.640
LMT	**0.529**	**74.47**	**0.769**	0.667
Logistic	0.512	**74.47**	0.760	**0.679**
NaiveBayes	0.351	65.96	0.692	0.600
NaiveBayesUpdateable	0.351	65.96	0.692	0.600
OneR	0.145	57.45	0.545	0.545
PART	0.183	59.57	0.537	0.579
RandomForest	0.191	59.57	0.578	0.565
RandomTree	0.065	53.19	0.522	0.500
REPTree	0.145	57.45	0.545	0.545
SGD	0.476	72.34	0.745	0.655
SimpleLogistic	**0.529**	**74.47**	**0.769**	0.667
VotedPerceptron	0.226	61.70	0.550	0.611
ZeroR	0.000	46.81	0.638	0.468

Table I: Classification performances of base classifiers for the AD dataset using the 18-protein biomarker.

**Classifier**	**MCC**	**Accuracy (%)**	**FMeasure**	**Precision**
BayesNet	0.848	89.13	0.886	0.786
DecisionStump	0.923	90.22	0.889	0.803
DecisionTable	0.923	90.22	0.889	0.803
IBk	0.930	**94.56**	**0.941**	**0.891**
J48	**0.951**	**94.56**	0.940	0.890
JRip	0.745	79.35	0.787	0.591
LibSVM	0.889	92.39	0.920	0.849
LMT	0.860	88.04	0.871	0.760
Logistic	0.837	85.87	0.847	0.716
NaiveBayes	0.891	93.48	0.932	0.873
NaiveBayesUpdateable	0.891	93.48	0.932	0.873
OneR	0.923	90.22	0.889	0.803
PART	0.946	90.22	0.886	0.806
RandomForest	0.864	89.13	0.884	0.783
RandomTree	0.791	81.52	0.800	0.628
REPTree	0.923	90.22	0.889	0.803
SGD	0.851	90.22	0.899	0.809
SimpleLogistic	0.860	88.04	0.871	0.760
VotedPerceptron	0.889	92.39	0.920	0.849
ZeroR	0.457	45.65	0.627	0.000

Table J: Classification performances of base classifiers for the MCI dataset using the 18-protein biomarker.

**Classifier**	**MCC**	**Accuracy (%)**	**FMeasure**	**Precision**
BayesNet	0.581	63.83	0.679	0.314
DecisionStump	0.545	57.45	0.545	0.145
DecisionTable	0.524	55.32	0.512	0.100
IBk	0.594	65.96	0.704	0.368
J48	0.571	59.57	0.558	0.186
JRip	0.594	65.96	0.704	0.368
LibSVM	0.613	68.08	0.717	0.404
LMT	**0.654**	**70.21**	0.708	0.414
Logistic	**0.654**	**70.21**	0.708	0.414
NaiveBayes	0.576	63.83	0.691	0.331
NaiveBayesUpdateable	0.576	63.83	0.691	0.331
OneR	0.545	57.45	0.545	0.145
PART	0.625	65.96	0.652	0.321
RandomForest	0.552	59.57	0.627	0.213
RandomTree	0.500	53.19	0.560	0.078
REPTree	0.545	57.45	0.545	0.145
SGD	0.633	**70.21**	**0.731**	**0.440**
SimpleLogistic	**0.654**	**70.21**	0.708	0.414
VotedPerceptron	0.619	63.83	0.605	0.272
ZeroR	0.468	46.81	0.638	0.000

Table K: Classification performances of base classifiers for the UAB datasets.

**Classifier**	**MCC**	**Accuracy (%)**	**FMeasure**	**Precision**
BayesNet	0.470	78.00	0.499	0.373
DecisionStump	0.321	80.80	0.273	0.212
DecisionTable	0.425	76.40	0.377	0.243
IBk	0.680	86.80	0.653	0.592
J48	0.420	76.80	0.435	0.293
JRip	0.547	81.20	0.554	0.443
LibSVM	**0.870**	86.80	0.541	0.530
LMT	0.863	**89.20**	**0.686**	0.646
Logistic	0.621	83.60	0.576	0.494
NaiveBayes	0.452	76.80	0.522	0.393
NaiveBayesUpdateable	0.452	76.80	0.522	0.393
OneR	0.344	76.80	0.294	0.166
PART	0.384	76.40	0.358	0.217
RandomForest	0.631	82.80	0.396	0.348
RandomTree	0.393	75.60	0.382	0.234
REPTree	0.468	77.20	0.464	0.328
SGD	0.760	88.00	0.646	0.591
SimpleLogistic	0.863	**89.20**	**0.686**	**0.646**
VotedPerceptron	0.603	84.00	0.585	0.491
ZeroR	0.000	80.00	0.000	0.000

Table L: Classification performances of base classifiers for the IAB datasets.

**Classifier**	**MCC**	**Accuracy (%)**	**FMeasure**	**Precision**
BayesNet	0.459	77.60	0.493	0.365
DecisionStump	0.321	80.80	0.273	0.212
DecisionTable	0.356	75.20	0.335	0.186
IBk	0.694	87.20	0.661	0.601
J48	0.454	78.40	0.452	0.322
JRip	0.392	74.40	0.437	0.280
LibSVM	**0.870**	86.80	0.541	0.530
LMT	0.821	**88.80**	**0.673**	0.626
Logistic	0.753	86.40	0.577	0.525
NaiveBayes	0.443	76.40	0.517	0.386
NaiveBayesUpdateable	0.443	76.40	0.517	0.386
OneR	0.344	76.80	0.294	0.166
PART	0.360	75.60	0.325	0.181
RandomForest	0.548	82.40	0.406	0.351
RandomTree	0.342	72.80	0.336	0.169
REPTree	0.468	77.20	0.464	0.328
SGD	0.824	**88.80**	0.666	0.624
SimpleLogistic	0.821	**88.80**	**0.673**	**0.626**
VotedPerceptron	0.589	83.60	0.601	0.501
ZeroR	0.000	80.00	0.000	0.000

Table M: Classification performances of base classifiers for the UEAB datasets.

Classifier	MCC	Accuracy (%)	FMeasure	Precision
BayesNet	0.533	81.20	0.569	0.456
DecisionStump	0.321	80.80	0.273	0.212
DecisionTable	0.416	77.60	0.337	0.223
IBk	0.678	86.40	0.649	0.572
J48	0.419	76.40	0.437	0.290
JRip	0.437	76.80	0.456	0.311
LibSVM	**0.894**	**88.80**	0.651	**0.629**
LMT	0.736	87.20	0.657	0.587
Logistic	0.610	80.00	0.472	0.384
NaiveBayes	0.458	76.40	0.536	0.412
NaiveBayesUpdateable	0.458	76.40	0.536	0.412
OneR	0.344	76.80	0.294	0.166
PART	0.464	78.00	0.476	0.342
RandomForest	0.696	84.40	0.528	0.464
RandomTree	0.388	75.20	0.356	0.209
REPTree	0.424	80.00	0.373	0.282
SGD	0.771	87.60	**0.667**	0.612
SimpleLogistic	0.736	87.20	0.657	0.587
VotedPerceptron	0.595	84.00	0.639	0.544
ZeroR	0.000	80.00	0.000	0.000
