# Supplementary material for: Heterogeneous Ensemble Combination Search Using Genetic Algorithm for Class Imbalanced Data Classification
Source: PLoS One. 2016 Jan 14;11(1):e0146116. doi: 10.1371/journal.pone.0146116 (PMC4713117; doi:10.1371/journal.pone.0146116)
Supplement: S2 File — This supporting information file describes about available features, system requirements and how to use the GA-EoC program. (PDF) [file pone.0146116.s002.pdf]

Supporting Information File S2

December 19, 2015

How to Use the Software

The GA-EoC: Genetic Algorithm-based Search Method for Heterogeneous Ensemble Classifiers has been implemented using Java programming Language. The source code is available at `https://sourceforge.net/projects/geneticensembleclassifier/` under GNU General Public License version 3.0 (GPLv3). The first beta release of the system uses pre-built CV datasets and their models for finding the best base classifiers combination to create the ensemble based on training dataset.

Required Libraries:

To compile and execute the program, it requires some software libraries. They are:

- **WEKA:** We use Waikato Environment for Knowledge Analysis (WEKA) version 3.7.10. The jar file for required version has been included in the "lib" directory. Alternatively, you can download it from publisher's website at `http://www.cs.waikato.ac.nz/ml/weka/index.html`
- **libsvm for WEKA:** For using the LibSVM classifier with weka, we have used Wrapper class for the libsvm library by Chih-Chung Chang and Chih-Jen Lin (`http://www.csie.ntu.edu.tw/~cjlin/libsvm/`). The original wrapper, named WLSVM, was developed by Yasser EL-Manzalawy (Yasser EL-Manzalawy (2005). WLSVM. `http://www.cs.iastate.edu/~yasser/wlsvm/`). We have included the required wrappers for using the LibSVM inside the "lib" directory.
- **Java:** we used JDK 1.6 for the development of the souce code. If you run the program in a multi-core computer, it will leave 2 processor cores free and use rest cores for this program.

Features:

This beta release of the system consists of following features:

- Generate Cross Validation folds and save datasets into disk for future usage in ARFF format

- Generate and serialise into disk of Classifier Models for all cross validation Training Folds for use by GA-EoC
- Generate and serialise into disk of All Base Classifier Models using the Full Training dataset.
- Search for best ensemble combinations to create heterogeneous ensemble of classifiers using k-fold cross validation on training dataset (using pre-generated CV dataset and models)
- Evaluate the performance of best ensemble combination on unknown Testing Data (use pre-generated models using full training data)

How to run the program:

To use the program please download the compressed file from the companion website. Follow these steps to run the program:

1. Unzip the GA-EoC-exe.tar.gz file into a directory.
2. To view available options and usage execute the program using “java -jar GE-EoC.jar”. It will show the help as Fig S2A.

Figure S2A: GA-EoC program showing the available options and usage while executing without ant commandline arguments.

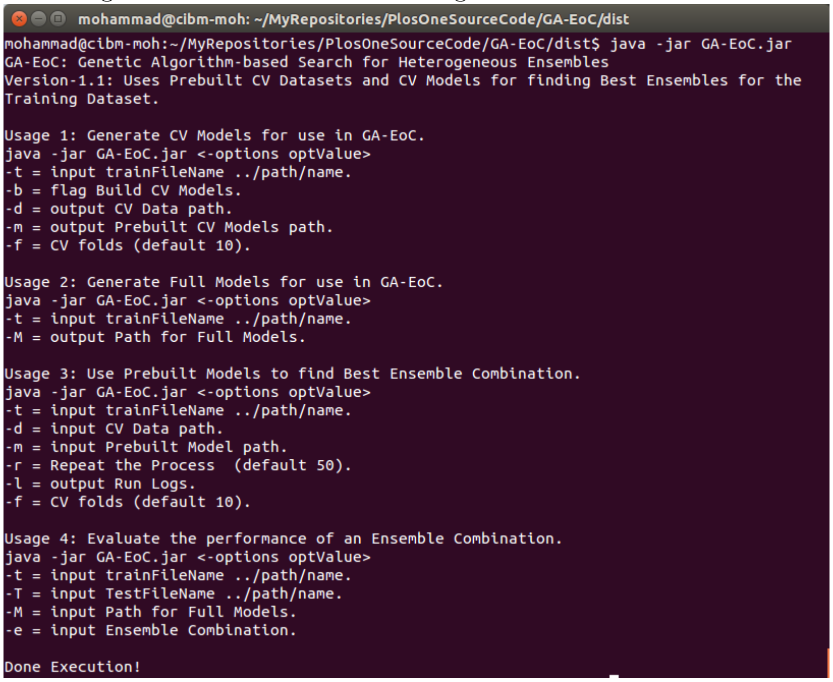

```
mohammad@cibm-moh: ~/MyRepositories/PlosOneSourceCode/GA-EoC/dist
mohammad@cibm-moh:~/MyRepositories/PlosOneSourceCode/GA-EoC/dist$ java -jar GA-EoC.jar
GA-EoC: Genetic Algorithm-based Search for Heterogeneous Ensembles
Version-1.1: Uses Prebuilt CV Datasets and CV Models for finding Best Ensembles for the
Training Dataset.

Usage 1: Generate CV Models for use in GA-EoC.
java -jar GA-EoC.jar <-options optValue>
-t = input trainFileName ../path/name.
-b = flag Build CV Models.
-d = output CV Data path.
-m = output Prebuilt CV Models path.
-f = CV folds (default 10).

Usage 2: Generate Full Models for use in GA-EoC.
java -jar GA-EoC.jar <-options optValue>
-t = input trainFileName ../path/name.
-M = output Path for Full Models.

Usage 3: Use Prebuilt Models to find Best Ensemble Combination.
java -jar GA-EoC.jar <-options optValue>
-t = input trainFileName ../path/name.
-d = input CV Data path.
-m = input Prebuilt Model path.
-r = Repeat the Process  (default 50).
-l = output Run Logs.
-f = CV folds (default 10).

Usage 4: Evaluate the performance of an Ensemble Combination.
java -jar GA-EoC.jar <-options optValue>
-t = input trainFileName ../path/name.
-T = input TestFileName ../path/name.
-M = input Path for Full Models.
-e = input Ensemble Combination.

Done Execution!
```

3. Use following commands depending on your intended usage:

   - **Usage 1:** Generate CV Models for searching best base classifiers combination.

     ```
     java -jar GA-EoC.jar <options optValue>
     -t = input trainFileName ../path/name.
     -b = enable flag Build CV Models (no values required).
     -d = output CV Data path.
     -m = output Prebuilt CV Models path.
     -f = CV folds (optional, default 10-fold cv).
     ```

   - **Usage 2:** Generate Full Models for evaluate ensemble combination.

     ```
     java -jar GA-EoC.jar <options optValue>
     -t = input trainFileName ../path/name.
     -M = output Path for Full Models.
     ```

   - **Usage 3:** Use pre-built CV Models to find the best combination of base classifiers to build the ensemble.

     ```
     java -jar GA-EoC.jar <options optValue>
     -t = input trainFileName ../path/name.
     -d = input CV Data path.
     -m = input Prebuilt Model path.
     -r = Repeat the Process (optional, default 50 repeatations).
     -l = output Run Logs.
     -f = CV folds (optional, default 10-fold cv).
     ```

   - **Usage 4:** Evaluate the performance of an ensemble of classifiers.

     ```
     java -jar GA-EoC.jar <options optValue>
     -t = input trainFileName ../path/name.
     -T = input TestFileName ../path/name.
     -M = input Path for Full Models.
     -e = input Ensemble Combination.
     ```

However, this program does not guarantee to be free from bugs. Bug reporting will highly appreciated for future development and stability of the program through the website.
